# Connecting high-resolution 3D chromatin maps with cell division and cell differentiation at the root apical meristem

**DOI:** 10.1007/s00299-024-03322-8

**Published:** 2024-09-16

**Authors:** Lara Caballero, Taras Pasternak, Riyazuddin Riyazuddin, José Manuel Pérez-Pérez

**Affiliations:** Instituto de Bioingeniería, Universidad Miguel Hernández, 03202 Elche, Spain

**Keywords:** Root apical meristem, Cell division, DNA replication, Cell differentiation, Euchromatin, Heterochromatin, Nuclear structure

## Abstract

**Key message:**

We used marker-free technologies to study chromatin at cellular resolution. Our results show asymmetric chromatin distribution, explore chromatin dynamics during mitosis, and reveal structural differences between trichoblast and atrichoblast cell.

**Abstract:**

The shapes, sizes, and structural organizations of plant nuclei vary considerably among cell types, tissues, and species. This diversity is dependent on various factors, including cellular function, developmental stage, and environmental or physiological conditions. The differences in nuclear structure reflect the state of chromatin, which, in turn, controls gene expression and regulates cell fate. To examine the interrelationship between nuclear structure, cell morphology, and tissue-specific cell proliferation and differentiation processes, we conducted multiple visualizations of H3K4me1, H3K9me2, 4′,6-diamidino-2-phenylindole, 5-ethynyl 2′-deoxyuridine, and SCRI Renaissance 2200, followed by subsequent quantitative analysis of individual cells and nuclei. By assigning cylindrical coordinates to the nuclei in the iRoCS toolbox, we were able to construct in situ digital three-dimensional chromatin maps for all the tissue layers of individual roots. A detailed analysis of the nuclei features of H3K4me1 and H3K9me2 in the mitotic and the elongation zones in trichoblast and atrichoblast cells at the root apical meristem revealed cell type-specific chromatin dynamics with asymmetric distribution of euchromatin and heterochromatin marks that may be associated with cell cycle and cell differentiation characteristics of specific cells. Furthermore, the spatial distribution of nuclei stained with 5-ethynyl 2′-deoxyuridine in the epidermis and cortex tissues suggests short-range coordination of cell division and nuclear migration in a linear sequence through an unknown regulatory mechanism.

**Supplementary Information:**

The online version contains supplementary material available at 10.1007/s00299-024-03322-8.

## Introduction

The nucleus of plant cells is a complex structure that plays a critical role not only in DNA storage but also in DNA replication, DNA repair, and regulation of gene expression in response to physiological and environmental signals (Goto et al. 2021). The principles of nuclear organization are conserved throughout evolution (Misteli 2020). The distribution of histones and histone marks, the organization of chromosomal territories, and the formation of higher-order chromosomal domains have been extensively studied using advanced confocal laser scanning, and super-resolution microscopy (Dumur et al. 2019; Amiad-Pavlov et al. 2021; Zhang et al. 2024). Several studies in different species, including the model plant *Arabidopsis thaliana*, have established three-dimensional (3D) models of chromatin organization (Pontvianne and Grob 2020). However, most of these studies have been performed on isolated nuclei, without taking into account cell types and positions.

Plant cells exhibit a great diversity of nuclear shapes, ploidy levels, and nuclear structural organization that varies among plant species and cell types within an organism. This diversity is influenced by cellular function, developmental stage, and physiological conditions (Carotenuto et al. 2019; Cantwell & Dey 2022). Differences in nuclear structure reflect the state of chromatin, which is known to regulate gene expression and thus cell fate (Mattout et al. 2015). Although these differences are known and have been described in previous work (Pavlova et al. 2022), they have not yet been precisely quantified at the whole organ level. Therefore, there is a growing need to better understand chromatin structure in plants in situ. Comparing nuclear structure in different plant species and at different developmental stages has the potential to reveal evolutionarily significant functional features of nuclear organization.

The INDEPTH project, which stands for Impact of Nuclear Domains on Gene Expression and Plant Traits, investigates the function of chromatin domains in nuclear processes and gene expression control (Tatout et al. 2022). Several semi-automated tools have been provided by the participating groups to characterize plant chromatin architecture, including NucleusJ (Desset et al. 2018), NucleusJ2.0 (Dubos et al. 2020), and NODeJ (Dubos et al. 2022). Standardized image acquisition and analysis procedures were developed to examine the spatial distribution of nuclear and chromosomal signals from 3D image stacks (Randall et al. 2022). Two complementary segmentation methods have been developed, one semi-automated (iCRAQ) and the other based on deep learning (Nucl.Eye.D). These methods allow the software to be trained with user-specific datasets and purposes, thus expanding the available toolbox in the field of plant cytogenetics (Johann to Berens et al. 2022). In addition, the 3D Object Counter plugin (Bolte and Cordelières 2006) allows the extraction of at least 25 parameters related to nuclear organization, including the quantification of ploidy level, chromatin abundance and distribution, nuclear shape, and nuclear asymmetry. The same plugin can be used to quantify euchromatin, heterochromatin, and DNA replication levels. In addition, the introduction of a spherical coordinate system for each individual nucleus may allow the study of their internal asymmetry in relation to its cell fate.

In our study, we applied nuclear DNA labeling in combination with whole-mount in situ localization of euchromatin and heterochromatin marks, quantitative analysis of DNA replication with 5-ethynyl 2´-deoxyuridine (EdU), and cell wall staining with SCRI Renaissance 2200 (SR2200) in each cell within the root apical meristem (RAM) of primary roots (PR) of young *A. thaliana* and *Solanum lycopersicum* seedlings. Subsequently, deep image analysis was performed to examine cellular morphology and nuclear organization in these root apices with 3D spatial resolution. Our experimental design uses conventional confocal laser scanning microscopy to examine the nuclear organization within the tissue context of whole organs. The objective of this study was to examine the dynamic regulation of euchromatin and heterochromatin in specific cell types within the proliferation domain of the RAM region. This approach recognizes the complexity and diversity of nuclear organization in plant organs and aims to capture it comprehensively. We examine here the diversity of nuclear and chromatin organization and its potential impact on nuclear and chromatin morphology, as well as DNA replication. Our results are consistent with the hypothesis that there is no universal model for nuclear organization and chromatin modification, and that variation exists between different cell types within plant organs and between species.

## Materials and methods

### Plant material and growth conditions

The plant genotypes used in these experiments were wild-type *A. thaliana* (L.) Heyhn. Columbia-0, FAN-GFP (Liu et al. 2023), and *S. lycopersicum* L. var. M82. Surface-sterilized *A. thaliana* seeds were distributed in square Petri dishes with TK1 medium (Pasternak et al. 2023) supplemented with 150 mg/L Bacto Tryptone (Roth, Karlsruhe, Germany), 1% sucrose, 5 mM (N- morpholino) ethanesulfonic acid (MES), and 1.1% agar (Roth) adjusted to a pH of 5.6. These plates were maintained at room temperature for 4 h to allow seed imbibition. After sowing, the plates were maintained at 4 °C for 12 h. The seedlings were cultivated on vertically oriented plates within a plant growth chamber at 22 °C under continuous white light. *S. lycopersicum* seeds were surface-sterilized in 25% hypochlorite for 30 min and sown in TK4 medium supplemented with 2% sucrose (Pasternak et al. 2021).

### 5-ethynyl 2´-deoxyuridine (EdU) labeling and immunolocalization

For labeling with EdU, three to five four-day-old *A. thaliana* seedlings were pre-incubated in TK1 liquid medium for 120 min in several vials as technical repeats, and further processed as described previously (Pasternak et al. 2022). In brief, 10 μM EdU (final concentration) was added to each vial at different time intervals (120, 310, and 355 min), and the seedlings were fixed in microtubule-stabilizing buffer (MTSB) 420 min after transfer to TK1 liquid medium. Following fixation of seedlings was carried out in a 4% formaldehyde solution in MTSB with a short vacuum pulse (2–3 min) followed by shaking for 45 min. Subsequently, the seedlings were treated with warm methanol (35–40 °C) for 15–20 min, followed by rehydration. Next, the seedlings were subjected to a series of treatments including cell wall digestion (35 min), membrane permeabilization (35 min), blocking with 2% BSA (30 min), primary antibody incubation (16 h at 4 °C), incubation with the EdU detection solution (45 min) with Alexa 647 as a marker, secondary antibody incubation (45 min), washing, and labeling with 0.3 mg/L of 4′,6-diamidine-2′-phenylindole (DAPI) or with SCRI Renaissance 2200 (SR2200) (dilution 1:10,000), before being mounted on a microscopic slide (Pasternak et al. 2022).

Immunolocalization was performed in *A. thaliana* Col-0 and *S. lycopersicum* M82 seedlings using a previously published whole mount in situ protocol (Pasternak et al. 2015). The affinity-purified anti-histone H3 antibodies utilized are detailed in Table 1. The Alexa-488/Alexa-555-conjugated anti-mouse or anti-rabbit secondary antibodies were diluted 1:400.

**Table 1:** Antibodies and dilutions used in this work

Antibodies	Host species	Clonality	Dilution used (WB; ICH)	Source	Product no
Anti-H3K4me1 (monomethyl Lys4)	Rabbit	Polyclonal	1:1000; 1:200	Abcam	ab1791
Anti-H3K4me2 (dimethyl Lys4)	Rabbit	Monoclonal	1:5000; 1:200	Abcam	ab176882
Anti-H3K4me3 (trimethyl Lys4)	Rabbit	Polyclonal	1:1000; 1:200	Agrisera	AS16 3190
Anti-H3K9me1 (monomethyl Lys9)	Rabbit	Polyclonal	1:1000; 1:200	Abcam	ab9045
Anti-H3K9me2 (dimethyl Lys9)	Mouse	Monoclonal	1:1000; 1:300	Abcam	ab1220
Anti-H3K9me3 (trimethyl Lys9)	Rabbit	Monoclonal	1:1000; 1:200	Abcam	ab176916
Anti-H3K27me1 (monomethyl Lys27)	Rabbit	Polyclonal	1:1000; 1:200	Sigma Aldrich	07–448
Anti-H3K27me3 (trimethyl Lys27)	Rabbit	Monoclonal	1:1000; 1:200	Sigma Aldrich	SAB5600009
Anti-H3K36me2 (dimethyl Lys36)	Rabbit	Polyclonal	1:1000; 1:200	GeneTex	GTX630555
Anti-H3K36me3 (trimethyl Lys36)	Rabbit	Polyclonal	1:1000; 1:300	Abcam	ab9050
Anti-H3K9Ac (acetyl Lys9)	Rabbit	Polyclonal	1:5000; 1:200	Sigma Aldrich	07–352

WB: Western blotting, ICH: immunohistochemistry

### Histone purification, ELISA, and Western blotting

Nuclear proteins were extracted from two-week-old seedlings of *A. thaliana* Col-0 and *S. lycopersicum* M82 under standard growing conditions by using the EpiQuik Total Histone Extraction Kit (EpigenTek, Farmingdale, NY, USA). The colorimetric assay EpiQuik Histone H3 Modification Multiplex Assay Kit (EpigenTek) was employed to screen for several histone H3 modification sites in the seedlings of *A. thaliana* Col-0 and *S. lycopersicum* M82 samples. This experiment employed an enzyme-linked immunosorbent assay (ELISA), in which several histone H3 modifications were captured simultaneously with their respective antibodies in a 96-well plate. Absorbance at 450 nm was then measured after colour development. Quantification and normalization were conducted in accordance with the manufacturer’s protocol. The purified nuclear proteins were separated on a 12% SDS–polyacrylamide gel and subsequently transferred to PVDF blotting membranes (GE HealthCare Technologies, Chicago, IL, USA) as indicated elsewhere (Kenesi et al. 2023). The PVDF membrane was incubated in the blocking solution overnight. The blocking solution employed was TBS-T (150 mM NaCl, 50 mM Tris–HCl, 0.2% Tween; pH 7.5) with 5% non-fat milk (Kenesi et al. 2023). Subsequently, the membranes were washed with TBS-T at least three times. Subsequently, the membranes were incubated with histone-specific primary antibodies (Table 1) for 4 h at room temperature. The membranes were then washed with TBS-T and incubated for 1 h with secondary antibodies (Goat-Anti-Rabbit-HRP: 1:10,000 [Agrisera, AS09602], Goat-Anti-mouse-HRP: 1:5000 [GE HealthCare, NA 931VS]) at room temperature. Subsequently, the immunoreactive signal was developed using WesternSure PREMIUM chemiluminescent substrate, and detection was performed using the ChemiDoc XRS + imaging system (Bio-Rad, Hercules, CA, USA).

### Image acquisition and processing

The TCS SP8 confocal microscope (Leica Microsystems, Mannheim, Germany) controlled by LAS X v3 software was used for image acquisition. The samples were imaged using a 20 × /0.75 air immersion objective for observation and an 86 × /1.20 water immersion objective (HC PL APO CS2) for image acquisition. Each acquisition consisted of a Z-stack capture of a single root with a 0.3−0.5 µm Z-slice distance, comprising 2–4 tiles. The excitation (Ex.) and emission (Em.) wavelengths used were as follows: DAPI and SR2200 (Ex./Em.): 405/424−503; Alexa 488 (Ex./Em.): 488/504−555; Alexa 555 (Ex./Em.): 555/558−643; Alexa 647 (Ex./Em.): 651/658−834. The images were converted to HDF5, stitched using the XUV tool, and saved as HDF5. The HDF5 files were utilized directly for root analysis or converted to TIFF for nuclei analysis.

### Nuclei/chromatin/replication analysis

Whole DNA staining was carried out by employing either DAPI or propidium iodide (PI) for nuclei labelling. Besides, the SCRI Renaissance 2200 (SR2200) or calcofluor white stain was also applied to visualize the cell walls (Robert et al. 2013). Nuclei detection and cell segmentation were performed according to a previous protocol (Pasternak and Pérez-Pérez 2021). Nuclei/chromatin analysis was conducted using the 3D Object Detection plugin in Fiji (Schindelin et al. 2012). The parameters analyzed for the selected nuclei included their relative XYZ position, volume occupied by the DNA, surface area, total fluorescent signal from the nuclei, nuclei width, height, depth (W, H, D) values, and relative distance between the center of mass and centroid, which were converted to Euclidian vectors based on the identified parameters in the 3D Object Counter plug-in (Bolte and Cordelières 2006) in Fiji (Schindelin et al. 2012). The measured dataset was saved as CSV files and exported to XLS files. The data was manually curated to exclude false positives (fused nuclei) and false negatives (background noise). The Cartesian coordinates were converted to cylindrical coordinates for the entire root, while individual nuclei were converted to spherical coordinates. Subsequently, cell fate was assigned to each nucleus based on its relative position along the longitudinal and radial axes of the root. Alternatively, the image file was exported to HDF5 and loading it into the iRocS toolbox (Schmidt et al. 2014; Pasternak et al. 2024). For the individual nuclei analysis, multiple nuclei sequences of the same type were selected, with each channel analyzed independently. The data were extracted according to the 3D Object Counter plugin (Bolte and Cordelières 2006) in Fiji (Schindelin et al. 2012).

To ascertain the extent of overlap between the DAPI and H3K9me1/2 signals, nuclei from a range of cell types and regions within the RAM were analyzed. The ImageJ program was employed to analyze each nucleus, selecting the equatorial plane, and creating a projection on the z-axis of a stack size. For each channel (DAPI and H3K9me1), a reference line is defined at the periphery of the nucleus. This enables the recovery of the coordinates and intensity values of the drawn line. To quantify the co-localization of the H3K4me1 and H3K9me2 signals, the ImageJ threshold color tool was employed. This tool allows for the differentiation of pixels with different color ranges within an RGB image. The nuclei of interest were isolated from the global image using tools such as the rectangle and the mirror. For the colocalization analysis, the composition of channels whose signal is of interest was maintained, and a range of colors was established to determine each region of interest. The total signal area is defined as ranging from 0 to 255, the exclusive red signal area is defined as ranging from 0 to 15, the signal area corresponding to the overlay is defined as ranging from 16 to 58, and the exclusive green signal area is defined as ranging from 59 to 255.

### DNA replication analysis

To analyze DNA replication, the labeled roots were sequentially scanned in four channels (nuclei, H3K4me3, H3K9me1/2, and EdU). To prevent saturation, the signal intensity in all channels was adjusted. Subsequently, the resulting images were exported to ImageJ, where the channels were split, and independent nuclei analysis was performed for each channel. The quantitative data pertaining to the level of DNA replication, as indicated by the integral EdU signal intensity for each nucleus, have been extracted. While in the case of heterochromatin replication, the intensity of each EdU spot was quantified for each nucleus.

## Results

### Establishing an experimental pipeline for a high-throughput study of chromatin structure in roots

**Fig. 1:**
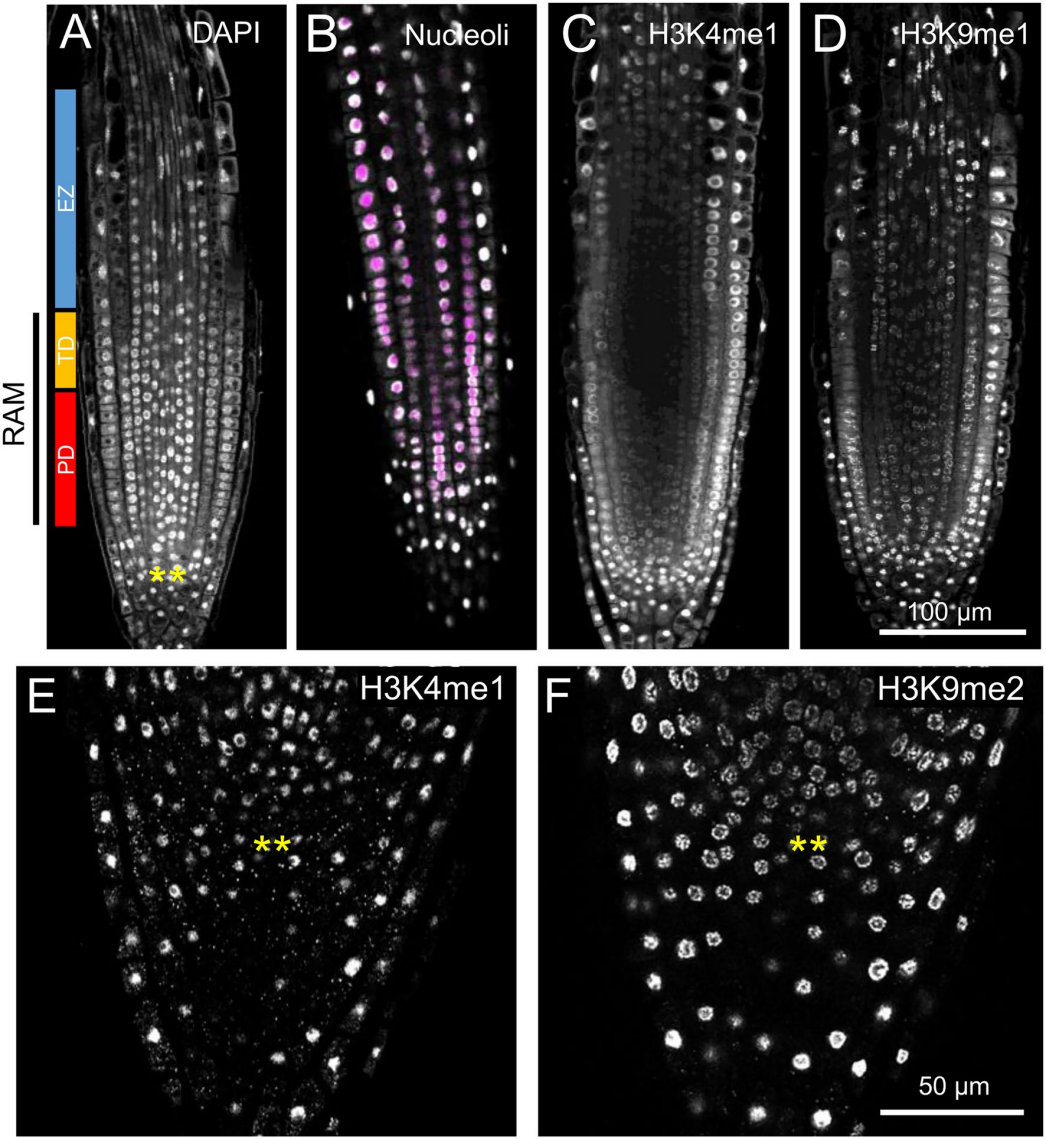
Methodology used to characterize nuclear heterogeneity in the RAM. **A** A representative *A. thaliana* primary root (PR) stained with DAPI for nuclei visualization, showing a longitudinal zonation pattern (Ivanov and Dubrovsky 2013). *PD* cell proliferation domain, *TD* transition domain, *EZ* elongation zone, *RAM* root apical meristem. **B** Nucleoli labeling with the FAN-GFP protein (magenta) and counterstained with DAPI (gray). **C**, **D** Immunolocalization was performed using the antibodies against the histone mark H3K4me1 (**C**) and H3K9me1 (**D**) in PRs of *A. thaliana*. **E**, **F** Detail of confocal images displaying the cells surrounding the QC that exhibit specific patterns of euchromatin (H3K4me1, E) and heterochromatin (H3K9me1, F). Yellow asterisks in **A**, **E**, and **F** indicate the quiescent center (QC) cells

An experimental procedure for DNA and cell wall staining was followed to characterize in detail the quantitative nuclear morphology and cell properties of root tips, including the identification of various euchromatin and heterochromatin marks. Serial stacks and tiles of the root tip from the columella and lateral root cap (LRC) cells to the elongation zone of the PRs, were obtained in both *A. thaliana* (Fig. 1) and tomato (Suppl. Fig. S1) seedlings. In the proliferation domain of the RAM region of both *A. thaliana* and tomato roots, nuclei exhibited intense DNA staining in discrete spots or chromocenters near the nuclear periphery (Fig. 1A, E and Suppl. Fig. S1). Moreover, no DNA staining was observed in the innermost region of the nuclei, which resembles the nucleoli. This was confirmed by the absence of overlap between the nucleolus-localized FAN-GFP fusion protein (Liu et al. 2023) and DAPI staining (Fig. 1A, B).

A total of 21 distinct histone H3 modifications were screened and quantified in nuclear extracts of young *A. thaliana* and tomato seedlings using a commercially available ELISA kit (Suppl. Fig. S2A). Some of the H3 modifications that have previously been correlated with different chromatin domains (Wang et al. 2015; Bi et al. 2017) were selected for further investigation (Table 1). In particular, H3K9me2/me3 is known to be associated with constitutive heterochromatin, whereas H3K4me3 and H3K4me1 are linked to euchromatin, given that they are involved in gene activation and transcription elongation, respectively (Hu and Du 2022), Among the H3 modifications that were tested, negative results were obtained for antibodies against H3K9me3, H3K27me3, and H3K36me2. Furthermore, the polyclonal antibodies against H3K27me1, and H3K36me3 showed non-specific bands of larger molecular weight and cannot be used for in situ maps as they produced a strong background during immunolocalization in root tissues (Suppl. Fig. S2B, C).

In our immunolocalization studies, H3K4me1, H3K4me2, H3K9me1, and H3K9me2 exhibited a consistent and precise nuclear pattern in all the roots studied for both species (Suppl. Fig. S2C). The euchromatic H3K4me1 mark (Fuchs et al. 2006) was observed to be homogenously distributed in all detectable nuclei within the *A. thaliana* and *S. lycopersicum* root tips, with no presence in the nucleolus (Fig. 1C, E). In contrast, H3K9me1 and H3K9me2 marks were observed to accumulate in discrete speckles around the nuclear periphery (Fig. 1D, E), which is a characteristic of heterochromatin (Baroux et al. 2007). The extent of colocalization between the H3K9me1, H3K9me2 signal and the chromocenters observed in DAPI staining was determined by comparing the signal intensity at the periphery of the central section of the nucleus. A strong colocalization between H3K9me1 and H3K9me2 was observed in the outer cell layers of the proliferation domain of the RAM region, irrespective of tissue type (Suppl. Fig. S3). Moreover, the chromatin staining profiles of adjacent nuclei of the same cell type in the proliferation domain of the RAM region revealed significant heterogeneity (Suppl. Fig. S3). Therefore, this nuclear heterogeneity as regards H3K9me1 and H3K9me2 may be indicative of the differentiation status of individual nuclei.

**Fig. 2:**
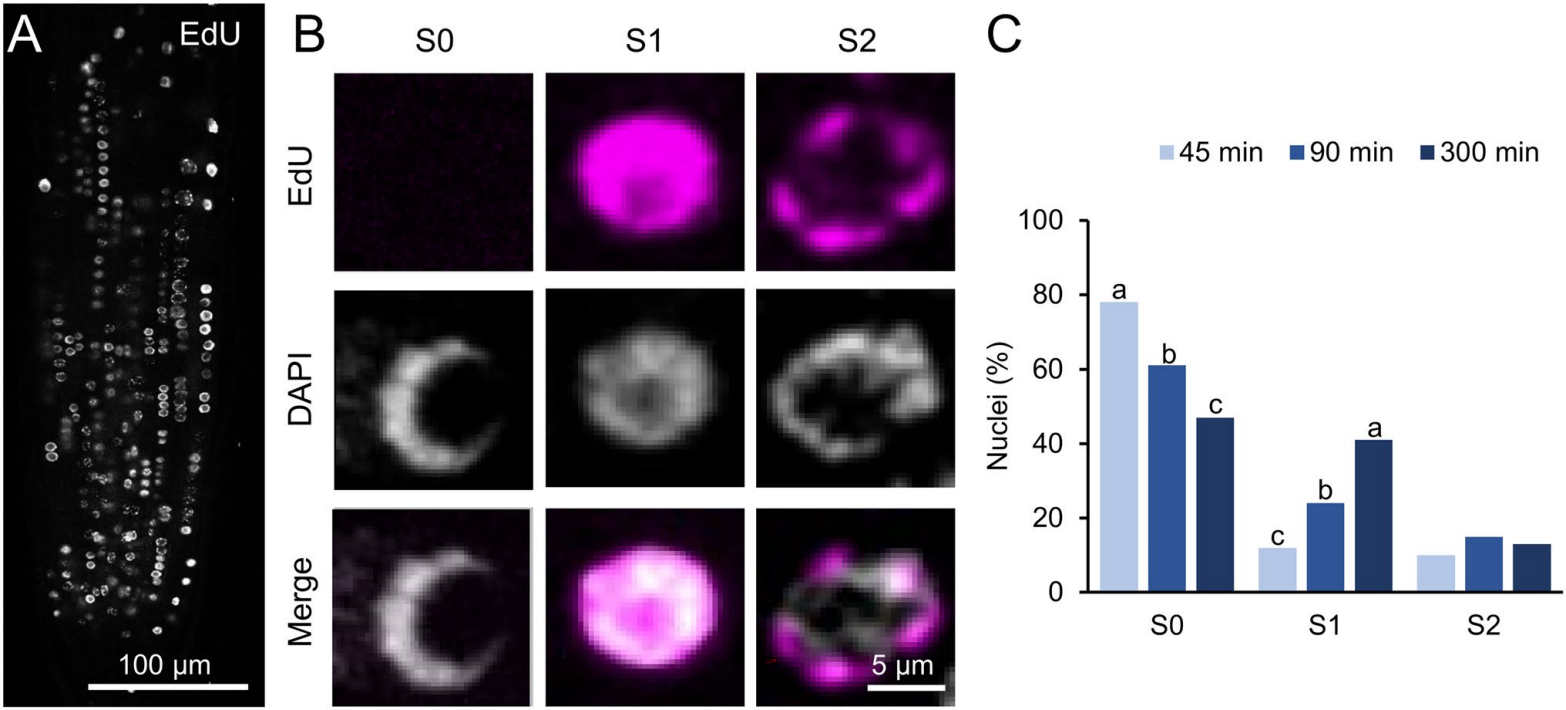
Detection of DNA replication in the RAM. **A** A representative *A. thaliana* root tip stained with EdU for measuring DNA replication. **B** Three EdU staining patterns were distinguished: S0, nuclei exclusively stained with DAPI; S1, homogeneous EdU staining; S2, characterized by the presence of EdU spots. **C** The study analyzed the proportions of S0, S1, and S2 nuclei at 45, 90, and 300 min of EdU incubation. Letters indicate statistically significant differences in the proportion of cells of each pattern depending on the incubation time with EdU (*P*-value < 0.05; Chi-squared test)

To ascertain the proportion of nuclei exhibiting replicating DNA, EdU labeling was employed at varying incubation times from 45 to 300 min, as described elsewhere (Pasternak et al. 2022). We focused both on the proliferation domain and the transition domain of the RAM region, which is where active DNA replication occurred. As an illustration, 45 min incubation with EdU is shown in Fig. 2A. A distinctive pattern of EdU staining was observed, extending from the proliferation domain to the transition domain of the RAM region (Pasternak et al. 2022). The data revealed the presence of multiple patterns of EdU incorporation. These included a homogeneous EdU staining pattern (S1), a spotted, incomplete EdU staining pattern that was colocalized with heterochromatin (S2), and combinations of both (Fig. 2B, C).

**Fig. 3:**
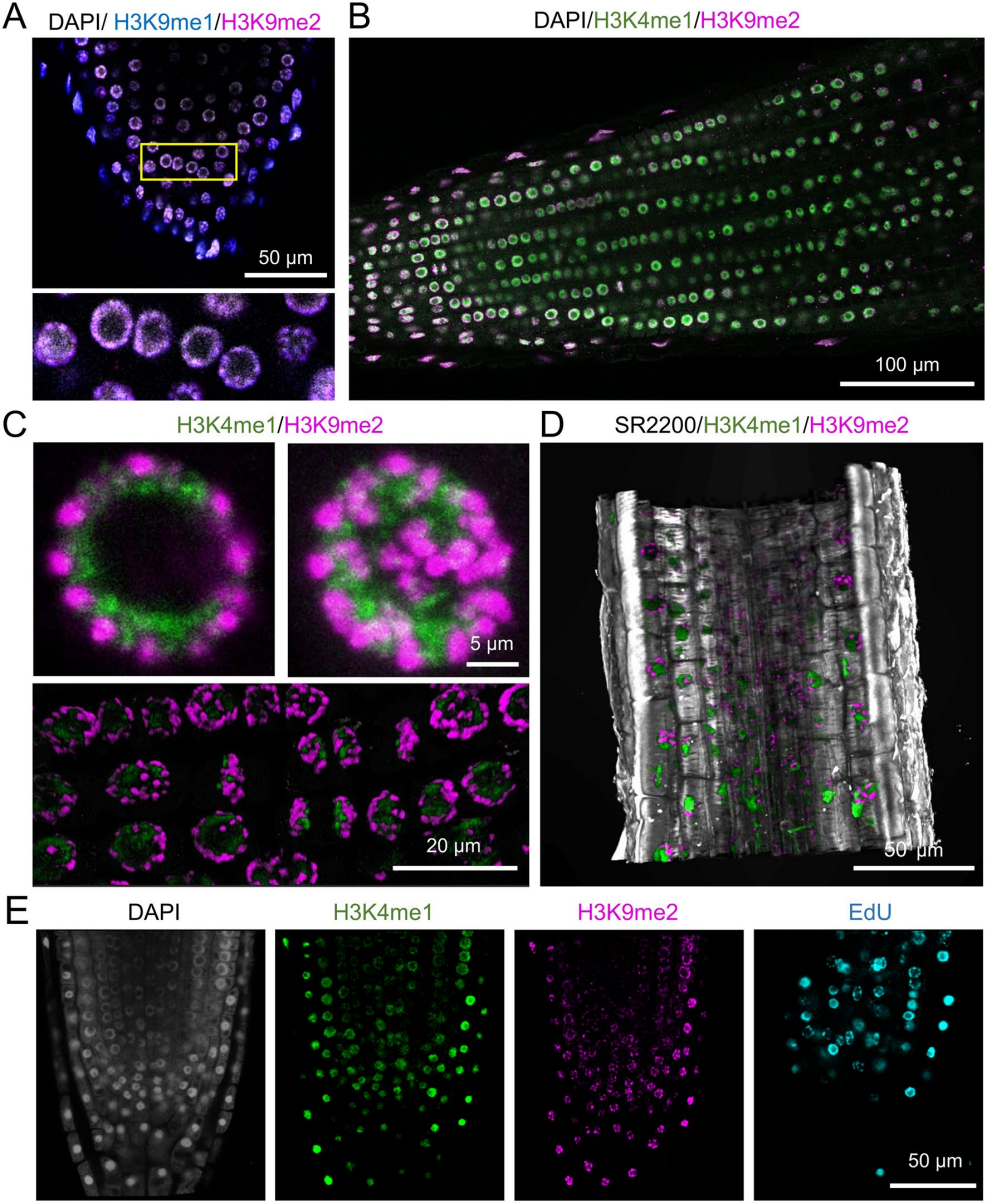
Multiple combinations for the characterization of nuclear heterogeneity. **A** Triple labeling of a tomato lateral root (LR) with DAPI (gray), H3K9me1 (cyan), and H3K9me2 (magenta). Lower panel is a magnification of the yellow inset from upper panel. **B** Triple staining with DAPI (gray), H3K4me1 (green), and H3K9me2 (magenta). **C** Double staining with H3K4me1 (green), and H3K9me2 (magenta). **D** Triple staining with SR2200 (gray), H3K4me1 (green), and H3K9me2 (magenta). **E** Quadruple staining with DAPI (gray), H3K4me1 (green), H3K9me2 (magenta), and EdU (cyan). A-D was performed in tomato LRs, and E was performed in *A. thaliana* PRs

We conducted double, triple and quadruple labeling with histone marks (H3K9me1, H3K9me2, H3K4me1) in conjunction with DAPI for DNA staining, SR2200 for cell wall visualization (Fig. 3 and Suppl. Movie 1), and EdU for visualization of DNA replication. The results of our analyses demonstrated that the triple labeling of H3K9me1, H3K9me2, and DAPI in tomato roots exhibited a tissue-specific localization pattern for heterochromatin marks in the LRC cells (Fig. 3A). Moreover, triple labeling with H3K4me1, H3K9me2, and EdU was successfully achieved for the entire *A. thaliana* RAM region (Fig. 3E). This approach enabled us to characterize the cellular morphology and nuclear heterogeneity in different cell types and throughout cell division with topological precision. Furthermore, subsequent studies were conducted on H3K9me2, given its precise localization to chromocenters in both species. We have elected to concentrate our efforts on *A. thaliana* seedlings, given their relatively low genome complexity and the extensive availability of genetic tools.

### Nuclear morphology and cellular parameters estimation along the axial dimension of the RAM

**Fig. 4:**
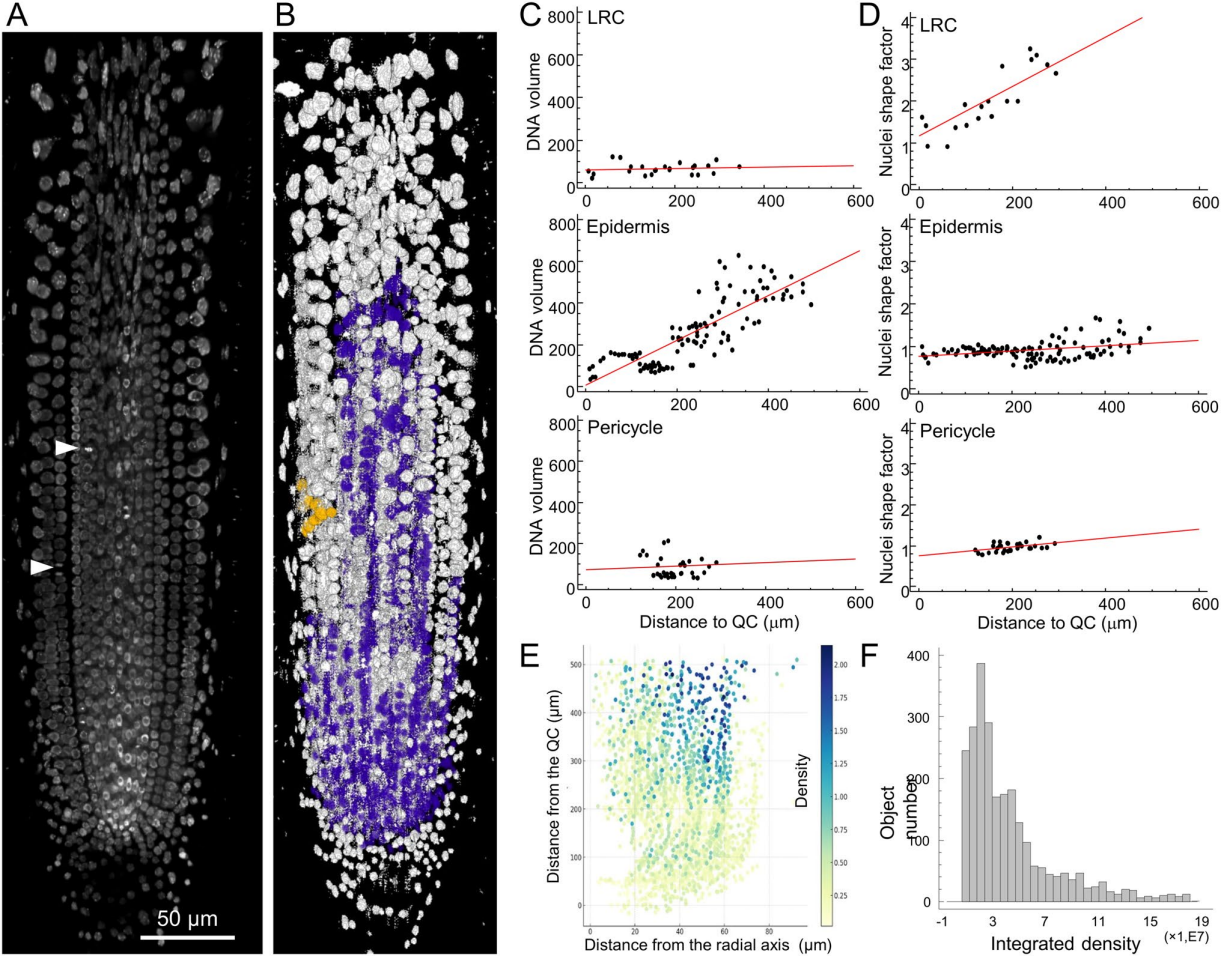
Nuclear parameter studies in different tissues. **A** A representative PR of *A. thaliana* stained with DAPI used for ploidy estimation. 3D view from 12 central sections is shown. White arrowheads show the position of the last mitosis of different tissues that define the proliferation domain (PD). **B** nuclei segmentation with 3D Object Counter plugin. **C** DNA volume in the indicated cell layers was estimated from the DAPI signal, and **D** the nuclei shape factor was determined by the width-to-height ratio of the fluorescent DAPI area. **E** In situ DNA labeling intensity map. About 2,500 nuclei were represented according to the radial axis of the root and their distance from the QC. The fluorescence of the DAPI signal is represented by the color intensity bar in the right side. **F** Frequency histogram based on DAPI fluorescence analysis of these nuclei

As illustrated in Fig. 1, there are notable differences in the morphology of the nuclei and the internal organization of chromatin among cell types within the RAM of *A. thaliana*, including the quiescent center (QC) region and the proliferation domain. To quantify these variations, a 3D-object analysis was conducted on all nuclei in single roots up to 600−700 µm (Fig. 4A, B). A regression analysis of the results from three representative cell types, including the LRC, epidermis, and pericycle, revealed significant differences in DNA volume and nuclear shape (Fig. 4C, D). The study discovered that the volume occupied by DNA of the LRC and pericycle cells remained stable, while the DNA volume of the epidermal cells increased progressively (Fig. 4C). Furthermore, the LRC nuclei elongated significantly along the root’s longitudinal axis, while the pericycle nuclei remained almost spherical (Fig. 4D). Moreover, based on the integral intensity of DNA labeling, we constructed maps of the relative nuclear ploidy distribution (Fig. 4E, F). A correlation was observed between the level of DNA fluorescence and the position of nuclei along the longitudinal axis of the root in the outer cell layers (Fig. 4E, F). This correlation defines the proliferation domain and the transition domain within the RAM region, thereby corroborating the findings of conventional ploidy studies but with otherwise spatial resolution.

**Fig. 5:**
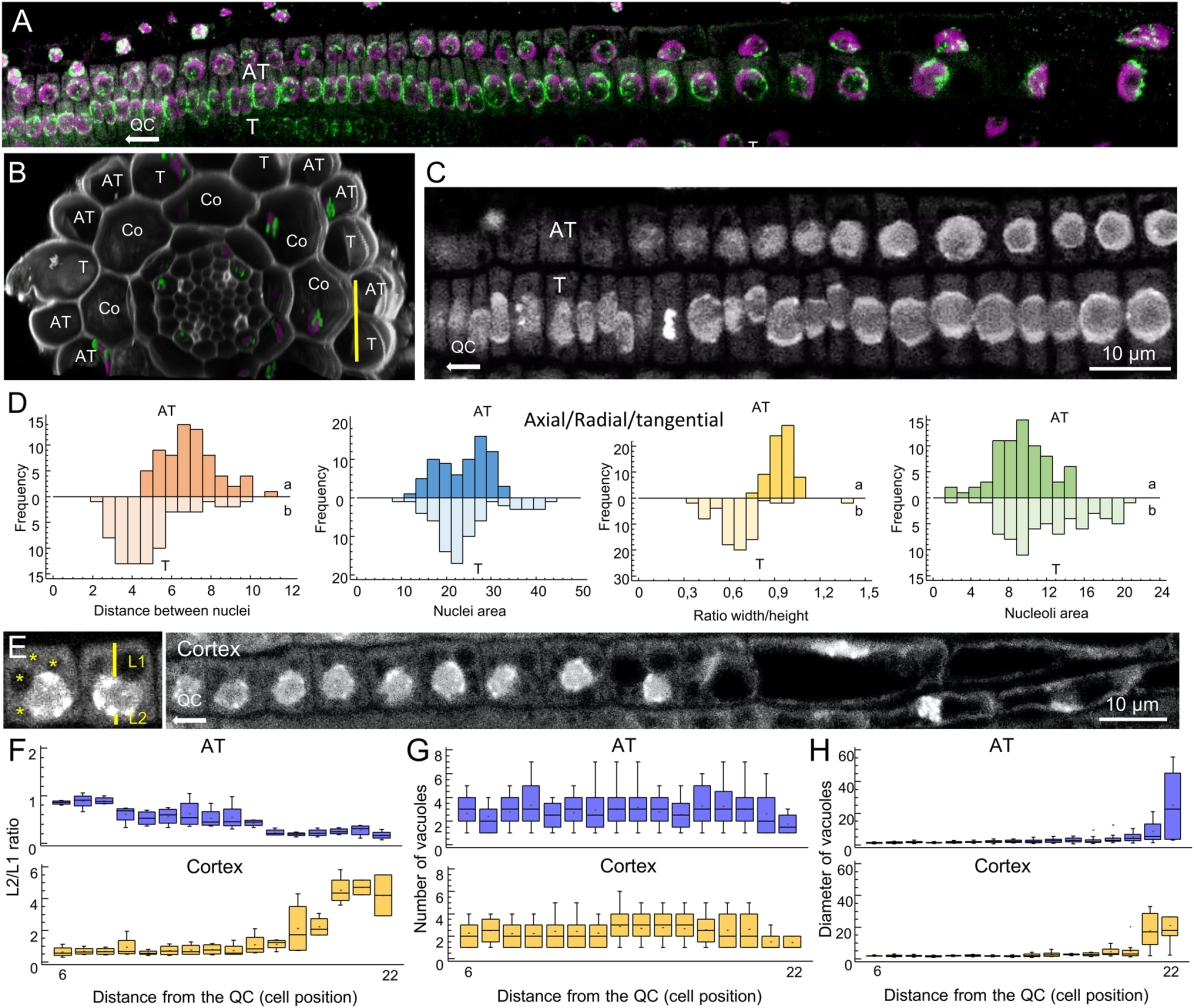
Spatial distribution of nuclear parameters in the root epidermis. **A** Triple labelling and staining with H3K4me1 (green), H3K9me2 (magenta), and DAPI (white) in a representative file of T and AT cells. **B**, **C** Spatial distribution of AT and T and their underlying cortical (Co) cells in a representative *A. thaliana* root; cross-section (**B**) and longitudinal view (**C**) of the proliferation domain (PD) and the elongation domain (EZ) of the RAM region. **D** Analysis of nuclear parameters related to nuclei shape and size in T and AT cells. **E–H** Morphometric analysis of subcellular parameters analyzed in cortex and overlying AT cells, including the relative position of the nucleus in the cell (**F**), measured by the distances from the edge of the nucleus to the outer cell wall (L1) and the inner cell wall (L2), as well as the number (**G**) and diameter (**H**) of vacuoles per cell (yellow asterisks). Letters in **F–H** indicate statistically significant differences between cell types (*P*-value < 0.01; LSD)

The root epidermis is composed of two distinct cell types, trichoblasts (T) and atrichoblasts (AT), which exhibit varying cellular attributes along the longitudinal and radial growth axes (Fig. 5A, B). In longitudinal confocal sections along the root surface covering the proliferation domain and the transition domain of the RAM region (Fig. 5C and Suppl. Movie 2), it was observed that T cells in the proliferation domain were approximately 1.8 times thinner than neighboring AT cells (Fig. 5D, distance between nuclei). Although the nuclei size in T and AT cells was similar (Fig. 5D, nuclei area), the nuclei of T cells exhibited a flattened morphology along the longitudinal axis of the root compared to those of AT cells. This was confirmed by the pronounced disparities in the shape factor values between T and AT nuclei (Fig. 5D, ratio width/height). The nucleoli area of T cells was found to be significantly larger than that of neighboring AT cells (Fig. 5D, nucleoli area).

At the proliferation domain of the root, the epidermal and cortex nuclei remained in a central position within the cell (Fig. 5E). However, as the nuclei proceeded through the transition domain and into the elongation zone, the epidermal nuclei migrated within the cell towards the center on the radial root axis, while the cortex nuclei moved away from it in an opposite, centripetal direction (Fig. 5F). The observed nuclear migration within epidermal and cortical cells may be dependent on vacuole morphology, as has been demonstrated for polar nuclear migration within T cells during root hair elongation (Nakamura et al. 2018). To test this hypothesis, we examined the number and size of vacuoles in AT and cortical cells located in the proliferation domain and the transition domain of the RAM region. It was observed that the number of vacuoles in both the AT and cortex cells was greater in the proliferation domain than in the transition domain (Fig. 5G). As the nuclei proceeded through the transition domain and into the elongation zone of the root, the number of vacuoles diminished, and their diameter increased (Fig. 5H). This observation is consistent with the migration patterns observed in the aforementioned cell types.

### Spatial distribution of euchromatin and heterochromatin marks during cell division and cell differentiation

**Fig. 6:**
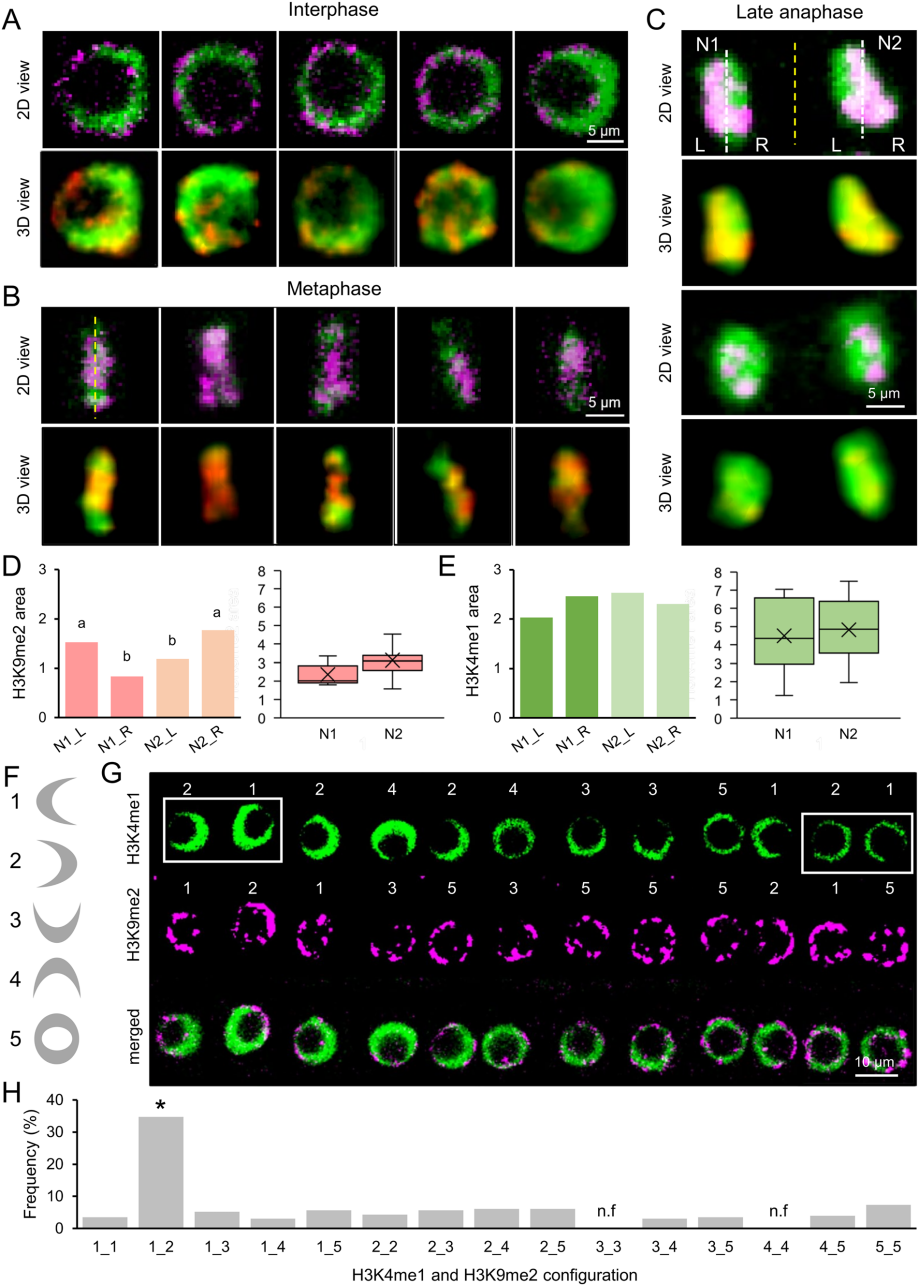
Distribution of H3K9me2 and H3K4me1 in AT cells. 2D and 3D views of several nuclei in interphase (**A**), metaphase (**B**), and late anaphase (**C**) are shown. H3K9me2 is shown in magenta/red, and H3K4me1 is depicted in green. Dashed yellow lines in **B** and **C** show the equatorial plane of the cell division. **D**, **E** The signal intensity for different chromosomic regions during anaphase was measured for H3K9me2 (**D**) and H3K4me1 (**E**). Letters indicate statistically significant differences between regions (*P*-value < 0.01; LSD). **F** Possible signal distributions considered in the individual nucleus for each mark. Numbers indicate the orientation of the signal in the axial direction. In H, the first digit indicates H3K4me1 orientation and the second, the orientation of H3K9me2. **G** A representative example of contiguous AT cells with an indication of the signal distribution patterns of H3K4me1 and H3K9me2 assigned. White squares indicate a contiguous nucleus that recently completed mitosis (1_2 pattern). **H** Frequency histogram of all possible H3K4me1 and H3K9me2 signal distributions in individual nucleus. The statistically significant differences are represented by the asterisk (*P*-value < 0.01; LSD); n.f.: not found (n = 230 nuclei, 3 roots)

The objective of this study was to investigate the dynamic regulation of euchromatin and heterochromatin marks during the mitotic stages in AT cells at the proliferation domain of the RAM region in *A. thaliana* seedlings. To achieve this, double staining with H3K4me1 and H3K9me2 was employed. A mere 6.1% of AT cells exhibited visible mitotic phases, spanning from metaphase to early telophase, within the proliferation domain of the RAM region. The analysis of 2D and 3D images of interphase nuclei (n = 170) demonstrated that the H3K9me2 mark is predominantly located in the peripheral region of the nucleus in the form of speckles of varying sizes, whereas the H3K4me1 mark exhibits a uniform distribution throughout the DNA and is excluded from the nucleolus (Fig. 6A and Supp. Movie 3). Although some degree of colocalization was observed between the two marks, the majority of their signal did not coincide (Fig. 6A and Suppl Fig. S4A).

At the metaphase plate, the H3K9me2 heterochromatin mark was observed to be internally located, while the H3K4me1 euchromatin mark was situated in a peripheral position in relation to it (Fig. 6B and Supp. Movie 4). Images captured during late anaphase revealed an uneven distribution of heterochromatin in the two daughter nuclei (Fig. 6C, Supp. Movie 5, and Suppl Fig. S4A), with a greater proportion of the H3K9me2 signal observed in regions situated further away from the equatorial plane of the mitotic plate (Fig. 6D). In contrast, H3K4me1 was distributed uniformly within the two daughter nuclei, exhibiting a slight increase in the signal in regions closer to the equatorial plane (Fig. 6E). It has been proposed that contiguous nuclei with opposing H3K4me1 and H3K9me2 signals along the longitudinal axis of the root, may indicate cells that have recently undergone mitosis. Given the heterogeneous distribution of H3K4me1 and H3K9me2 signals across contiguous AT nuclei, we have devised a nomenclature to delineate the spatial relationship between these two marks in individual nuclei (Fig. 6F). It was postulated that the probability of occurrence for all potential configurations of uneven H3K4me1 and H3K9me2 in single nuclei would be equal if a random chromatin rearrangement occurred following mitosis. However, we observed a higher proportion than expected of interphase nuclei with the opposing distribution of H3K4me1 and H3K9me2, which we have designated the 1_2 configuration (Fig. 6G, H). Accordingly, this configuration indicates that H3K4me1 (euchromatin) and H3K9me2 (heterochromatin) were in opposite compartments of the nucleus. This suggests that the opposite euchromatin and heterochromatin distribution (1_2 configuration) may be more stable in interphase AT nuclei. It is noteworthy that chromatin configurations in which H3K4me1 and H3K9me2 signals colocalize completely in regions orthogonal to the equatorial plate of cell division (designated as 3_3 and 4_4 configurations), were not observed (Fig. 6H). In conclusion, the data indicated that there was a degree of chromatin asymmetry in the nuclei, with a clear separation of euchromatin and heterochromatin marks on opposite sides of the nucleus shortly after mitosis.

A significant difference was identified in the colocalization degree between H3K4me1 and H3K9me2 marks in T and AT cells within the proliferation domain of the RAM region (Suppl. Fig. S4B, C). Notably, the H3K9me2 signal exhibited a higher degree of variability in nuclei at the upper elongation zone and adjacent to the differentiation zone of the root when compared to the H3K4me1 signal in this region (Suppl. Fig. S4D–F and Suppl. Movie 6). The ratio of H3K9me2 to H3K4me1 is higher in the outer radial layers, such as the epidermis and cortex, compared to the internal cells of the pericycle and vascular region, which have a lower ratio of H3K9me1 to H3K4me1 (Suppl. Fig. S4G–I). This may be regarded as an epigenetic signature of the transcription of genes associated with the topology of individual cells within the root tip. Such significant differences in euchromatin topology led to epigenetic and geometric constraints and serve as a reason for root abnormalities under different environmental perturbations, such as those evidenced by carbon starvation of the PRs (Supp. Fig. S5). An intriguing tissue-specific pattern of H3K9me2-enriched heterochromatin was observed in the elongation zone of the RAM. In this region, the levels of H3K9me2 were significantly higher in the epidermis and cortex cells and markedly lower in the inner tissues of the pericycle and vascular cells. These distinctive features, including nuclear enlargement and large-scale chromatin rearrangements, are consistent with cells that have exited the cell cycle exit prior to terminal differentiation. However, this remodeling was not observed in the internal cell layers, including the pericycle, which exhibited a lower ratio of H3K9me2 to H3K4me1 in their nuclei. These findings are in accordance with the hypothesis of a dome-shaped structure of the RAM, whereby the outer layers undergo differentiation at a higher rate than the inner tissues, as previously proposed (Lavrekha et al. 2017).

### Staining of DNA replication as a tool to measure cell cycle synchronization

**Fig. 7:**
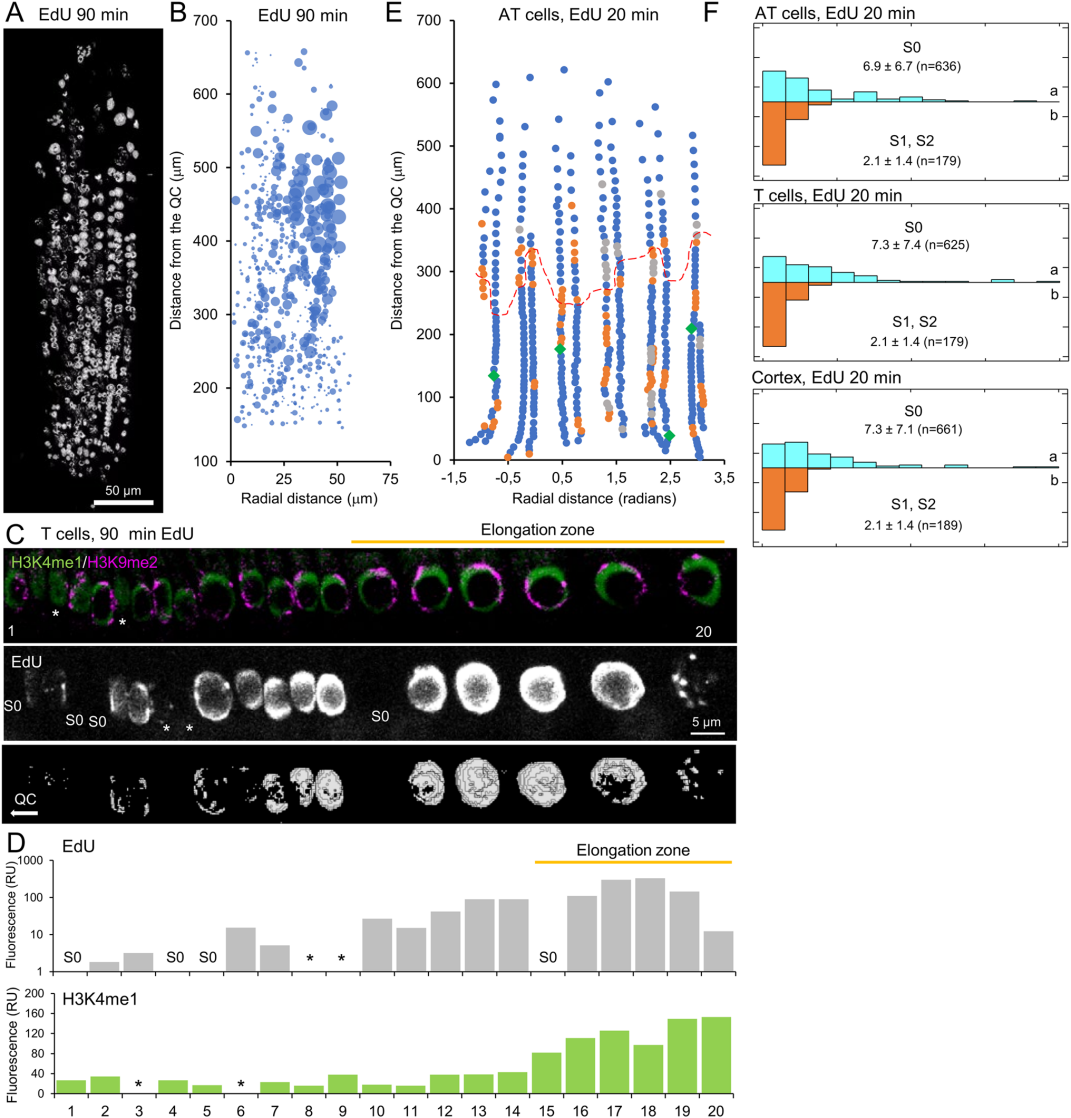
Quantification of DNA replication in the RAM. **A** A representative *A. thaliana* root tip used for the quantification of DNA replication. Seedlings were labelled with EdU for 90 min, scanned with a confocal microscope, stitched and subjected to 3D segmentation. **B** The analyzed nuclei were represented along the longitudinal and radial growth axes. Bubble size indicates the intensity of fluorescence per nuclei. **C** Triple labelling and staining with H3K4me1 (green), H3K9me2 (magenta), and EdU (white) in a representative file of T cells along the region between the PD and the EZ, dubbed as the transition domain (TD). The third panel represents the results from EdU segmentation. White asterisks indicate nuclei that were not correctly segmented or with low/no fluorescence signal. **D** Histograms with relative fluorescence of the EdU (grey, logarithmic scale) and H3K4me2 (green, linear scale) on the nuclei depicted in C. S0 refers to cells without EdU staining. Orange lines in **C** and **D** mark cells in the EZ. **E** A representative unrolled root epidermis showing AT nuclei in S0 (blue), S1 (orange), and S2 (grey) patterns. Nuclei with mitotic figures are depicted as green diamonds. The orange dashed line indicates the PD/TD boundary, which was defined by cell size increase. **F** The distribution of clusters of nuclei in the analyzed tissues of AT cells (top) and cortex cells (bottom) for the S0 and S1/S2 patterns of EdU staining are represented in histograms. Letters indicate significant differences between grouping in S0 and S1/S2 nuclei

To evaluate DNA replication patterns, EdU was employed on *A. thaliana* roots following distinct incubation periods from 45 to 300 min (Suppl. Fig. S6A). Nuclei were classified based on the distinct EdU staining patterns observed as homogeneous EdU staining pattern (S1) and spotted, incomplete EdU staining pattern (S2) (Fig. 7A and Supp. Movie 7). Following a brief 45-min incubation period with EdU allowed us to visualize only cells during DNA replication or shortly after (Pasternak et al 2022). It was observed that between 10% (LRC cells, n = 568) and 20% (epidermis [n = 297], and cortex [n = 122] cells) of the nuclei exhibited EdU staining (Suppl. Fig. S6B). Additionally, mitotic chromosomes were observed in only 1.6% of the analyzed nuclei (*n* = 987). Following an extended incubation period with EdU (300 min), the proportion of stained nuclei increased to 60% in epidermis (*n* = 483) and cortex (*n* = 203) tissues, while in LRC cells (*n* = 621), the proportion reached 30% after 300 min of EdU incubation (Fig. 7A and Suppl. Fig. S6A, B). Moreover, the proportion of nuclei undergoing mitosis exhibited variability across different cell types, with 0.63% in LRC (*n* = 1,595) and 1.58% in cortex (*n* = 569), indicating a differential division rate between tissues, as previously reported (Pasternak et al. 2022). In these conditions, a progressive increase in the proportion of nuclei in pattern S1 was observed in all cell types from 45 to 300 min of EdU incubation. However, the proportion of nuclei in pattern S2 remained constant even after prolonged incubation with EdU (Suppl. Fig. S6B). This suggests that pattern S1 identifies nuclei in the early S phase, whereas pattern S2 identifies nuclei in the late S phase. Furthermore, to ascertain which type of chromatin replicates DNA during the early or late S phase, we conducted an analysis of the colocalization of the H3K4me1 or H3K9me2 marks with the S1 and S2 patterns of EdU in the epidermis (Suppl. Fig. S6C, D). In both T and AT cell types, a greater degree of colocalization of the euchromatin mark H3K4me1 was observed in the nuclei of the pattern S1, whereas the pattern S2 exhibited a greater degree of colocalization with the H3K9me2 mark, which is characteristic of heterochromatin (Suppl. Fig. S6E, F). These findings align with the proposed hypothesis that DNA replication initiates concurrently in broad euchromatic regions within the nucleus during the early S phase (S1 pattern), while heterochromatin replication occurs later (S2 pattern) (Costas et al. 2011; Concia et al. 2018).

We postulated that the fluorescence intensity of H3K4me1, H3K9me2, and EdU could be employed to estimate different parameters (volume, surface, etc.,) of euchromatin, heterochromatin, and replicating DNA, respectively, in individual nuclei with spatial resolution. Following a 90-min period of EdU incubation, a notable variation in EdU fluorescence was observed along the longitudinal and radial growth axes of the root tip (Fig. 7B). To further investigate the relationship between chromatin structure and DNA replication, we conducted triple staining after 45 min of incubation with EdU, in defined regions of the RAM. The quantification of the EdU signal was employed to identify nuclei that were not engaged in DNA replication (i.e., exhibiting the S0 pattern) and to determine the extent of DNA replication in neighboring nuclei. In a representative sample of T nuclei from the region between the proliferation domain and the elongation zone, we observed that H3K4me1 levels were similar in the proliferation domain region and increased 2.5-fold in the elongation zone (Fig. 7C, D), which is consistent with the observed increase in ploidy levels in this cell type (Fig. 3A). Regular patterns and levels of EdU staining were observed in cells in the proliferation domain, with contiguous cells displaying the same pattern and similar levels of fluorescence localization. In the elongation zone, nuclei mostly exhibited high levels of EdU signal (Fig. 7C, D), which colocalize with euchromatin, indicating coordinated entry into the endoreduplication phase. Following a brief pulse labeling with EdU, patches of nuclei with similar staining patterns were predominantly observed along longitudinal cell files, including the epidermis and cortex (Fig. 7E). The nuclei that were not stained with EdU (pattern S0) exhibited an inverse Gaussian distribution in both tissues analyzed, AT cells and cortex cells (Fig. 7F), with no significant differences between them (*P*-value = 0.532). In contrast, the nuclei stained with EdU were most frequently observed in groups of one (42.2%) or two (31.8%) nuclei, with no significant differences between AT and cortex nuclei (*P*-value = 0.916). No significant differences were observed in the grouping pattern of non-EdU stained nuclei between the proliferation domain and the elongation zone in any of the analyzed tissues (*P*-value = 0.118). The average number of EdU-stained nuclei was 2.3 in the proliferation domain and 1.7 in the elongation zone for both tissues (*P*-value = 0.012). A comprehensive examination of the alterations in nuclear morphology and the distribution of euchromatin and heterochromatin marks in diverse cell types within the RAM has enabled us to discern significant discrepancies in the growth and nuclear organization of cells in adjacent layers. Furthermore, the intracellular migration of their nuclei suggests the potential existence of regulatory mechanisms that govern these processes. Our long-term objective is to adapt this approach to other crops to elucidate the dynamics of chromatin maps and the subsequent impact on gene expression associated with development and stress tolerance.

## Discussion

The present work outlines a methodology for the establishment of an in situ chromatin profile with cellular resolution. The procedure entailed staining the cell border, nucleus, euchromatin, and heterochromatin, as well as the detection of DNA replication events. The combination of a cylindrical coordinate system with the iRoCS toolbox (Schmidt et al. 2014) and graphical visualization tools like ggPlantmap (Jo and Kajala 2024) enables the creation of digital 3D maps of the RAM, encompassing cellular, nuclear, and chromatin features for each cell.

Histone modifications, including acetylation, methylation, and phosphorylation, exert a direct influence on the structure of chromatin and, consequently, on nuclear morphology. This influence is exerted either directly or by regulating the binding of chromatin-associated factors (Bannister and Kouzarides 2011; Tessarz and Kouzarides 2014). The present study has confirmed the specificity of several chromatin methylation marks in H3K4 and H3K9 in the roots of *A. thaliana* and tomato. Two of these histone modifications are associated with heterochromatin while the other two are associated with euchromatin. The H3K4me1 mark was asymmetrically distributed throughout the nucleus, exhibiting a dense and highly organized distribution in the proliferation domain of the RAM region and being absent from the nucleoli in all studied cells. This finding is consistent with its known location in active gene enhancers situated within the euchromatic regions of the genome (Zhang et al. 2009). The chromocenters observed in the DAPI staining of the nuclear DNA were found to correspond to the heterochromatin regions marked with H3K9me1 and H3K9me2 (Fig. 5). Chromocenters are typically situated in the outermost region of the nucleus, in close proximity to the nuclear envelope. A noteworthy diversity in chromocenter number, size, and spatial distribution was observed across different cell types and along the longitudinal axis of the root, which were found to be associated with specific cell fate and/or cell cycle stages. Specifically, the H3K9me2 signal displayed increased intensity in the outer cell layers of both *A. thaliana* and tomato roots.

H3K9me2 is a conserved mark of peripheral heterochromatin that is closely associated with the nuclear lamina in animal cells (Poleshko et al. 2019). In *A. thaliana*, the H3K9me2 mark is present in extensive blocks of pericentromeric heterochromatin, as well as in specific regions comprising euchromatin repeats and transposons (Roudier et al. 2011; Ramakrishnan Chandra et al. 2024). In interphase nuclei, the H3K9me2 heterochromatin mark is located in specific speckles along the peripheral region of the nucleus and in close proximity to the nuclear envelope, with minimal overlap with the euchromatin H3K4me1 mark (Pei et al. 2021). The distribution of the latter is homogeneous within the interphase nucleus. The observed dynamic rearrangement of H3K9me2 and H3K4me1 during metaphase and anaphase is consistent with the hypothesis that H3K9me2-enriched pericentromeric heterochromatin plays a role in anchoring the mitotic spindle during mitosis (Yamada and Goshima 2017; Ramakrishnan Chandra et al. 2024). During metaphase, the mitotic spindle assembles with centromeric heterochromatin. In late anaphase, kinetochore microtubules undergo a further shortening, which contributes to the separation of heterochromatin towards the poles of the equatorial plane of mitosis. Euchromatin, which is less condensed, remains in the region opposite to that of the condensed chromatin, situated in close proximity to the equatorial plane. Following the conclusion of mitosis and the reconstruction of the nuclear envelope, the two contiguous daughter cells exhibit concave and convex patterns, respectively, of H3K4me1 nuclear distribution (white square in Fig. 6G). Moreover, following the formation of the cell plate, H3K9me2 exhibited contrasting distribution patterns (1_2 configuration). Our study identified approximately 40% of AT nuclei exhibiting this pattern, indicating that these cells have completed mitosis and are in the early G1 phase. It is noteworthy that the spatial distribution patterns of the H3K4me1 and H3K9me2 marks in the remaining 60% of the analyzed AT nuclei exhibited an independent and random distribution, with the exception of the absence of patterns in which these two marks were present in the region of the nucleus closest to the T cells. These findings suggest the possibility of spatial constraints on chromatin distribution between cells across different rows, but not within the same row. Further experimentation is required to extend this observation to other tissues, including the cortex and endodermis.

The relative DNA content of each nucleus can be estimated from the provided dataset by integrating the H3K4me1 signal and/or DAPI fluorescence data. It was assumed that the nuclei of the initial cells surrounding the QC exhibited a DNA level of 2C (Bhosale et al. 2018), which can be used as a reference for estimating the ploidy levels of the other nuclei under study. Cylindrical coordinates were assigned to individual nuclei in the iRoCS toolbox (Schmidt et al. 2014) for the purpose of constructing in situ ploidy maps for all the tissue layers within a single root. The maps can be utilized to characterize known mutants affected in endoreduplication and cell cycle progression, such as *kip-related protein 5* (Jégu et al. 2013) and *cyclin D3* triple mutants (Dewitte et al. 2007). The DNA volume and shape factor of the nucleus exhibited variation depending on the cell type and differentiation status along the longitudinal root axis. The nuclei of differentiating LRC cells underwent a transformation from a spherical shape with homogeneous DNA distribution to an elongated shape with marked chromocenters, accompanied by minor fluctuations in the amount of DNA per nucleus. In contrast, the nuclei observed at the root epidermis exhibited a notable increase in DNA levels, extending beyond the RAM region of the root. This observation is consistent with the endoreduplication of DNA that occurs in this region for the epidermal cells and that precedes their rapid cell expansion at the elongation zone of the root (Hayashi et al. 2013).

Mitosis is a relatively brief process, resulting in a low percentage of cells undergoing mitosis within the RAM region at a given time (Pasternak et al. 2022). By incubating young seedlings with EdU for increasing periods of time, we were able to conduct a comprehensive examination of the dynamics of DNA replication during the S-phase of the mitotic cell cycle and the endocycle at a cellular resolution in the RAM. This process is dependent on the activation of the replication origins through ORC1b (Vergara et al. 2023). The staining patterns observed for EdU, which are homogeneous (S1) and spotted (S2), suggest that DNA replication initiates concurrently in broad euchromatic regions within the nucleus during the early S phase (S1 pattern), while heterochromatin replication occurs later (S2 pattern) (Costas et al. 2011; Concia et al. 2018). These findings align with the proposed hypothesis, as evidenced by a stronger overlap between H3K9me2 and EdU signals in the S2 pattern, and a higher degree of overlap of H3K4me1 with EdU in the S2 pattern. The relative abundance of EdU over H3K4me1 was quantified to assess the extent of DNA replication occurring during the mitotic cell cycle and the endocycle. The observed differences in the pattern of EdU-stained nuclei of cells at the transition domain suggest the existence of tissue-specific S-phase durations, which require further investigation.

In the roots of *A. thaliana* and tomato, the outermost layer of the epidermis is composed of alternating T and AT cells with distinct geometry and cell fate (Berger et al. 1998). T cells are radially connected to two inner cells of the exodermis layer (in the case of the tomato) or the cortex layer (in the case of *A. thaliana*), while AT cells are connected to a single underlying exodermal or cortical cell. In the EZ, the number of T cells in each longitudinal row is approximately twice that of AT cells. The nuclei of T cells are flattened along the longitudinal axis of the root and exhibit larger nucleoli than those of AT cells, which may result in elevated mechanical stress on the nuclei of the T cells. In both T and AT cells within the proliferation domain of the RAM region, the nuclei are positioned centrally within the cell. These observations are consistent with the hypothesis that T cell undergo a higher frequency of formative divisions than AT cells (Berger et al. 1998). In consideration of the three-dimensional structure of the epidermis and the subepidermal layer, it becomes evident that a supracellular mechanism is indispensable for the coordination of the expansion of AT cells and their underlying cortex/exodermis cells with the cell division of the T cells with which they are in contact. It has been proposed that in root cells, brassinosteroid-mediated microtubule arrangement regulates cell shape through differential regulation of directional growth between the inner and outer tissues (Fridman et al. 2021). Given that *brassinosteroid insensitive 1* mutants exhibit a reduced number of divisions in the epidermis (Fridman et al. 2021), it is imperative to conduct a comprehensive examination of the cellular and nuclear characteristics of T and AT cells in these mutants to elucidate the mechanism of growth coordination in roots.

In plant cells, the nuclei undergo a change in position in response to alterations in the surrounding environment, the polar growth of specific cells, and during cell division. External factors, such as the direction of light or the presence of other cells, have been demonstrated to influence this positioning (Wada 2018). In the leaves of *A. thaliana*, the nuclei of mesophyll cells are observed to relocate to the side walls following continuous exposure to intense blue light. This relocation is proposed to serve as a protective mechanism against ultraviolet (UV)-induced DNA damage (Iwabuchi et al. 2016). The polar nuclear movement observed during root hair growth in T cells at the differentiation zone of the root is driven by an as yet unidentified actin-based mechanism that is dependent on auxin signaling (Nakamura et al. 2018; Brueggeman et al. 2022). Our study revealed a previously unidentified phenomenon: the migration of the nuclei of AT cells and cortex cells in the transition domain of the *A. thaliana* roots. The results indicate that AT nuclei rapidly migrate towards the inner side of the cell, while nuclei in the cortex cells exhibited a centripetal movement upon reaching the transition domain of the RAM. It is conceivable that the concomitant migration of AT and subjacent cortex nuclei may facilitate the coordination of growth responses among neighboring cells through the mediation of specific short-range non-cell autonomous signals via the plasmodesmata. The results of our study indicate that vacuole biogenesis may be a crucial factor in the migration of AT and cortex nuclei. The *shoot gravitropism 2* mutant, which displays abnormalities in its vacuolar structure, exhibited impeded nuclear migration during zygote polarization (Kimata et al. 2019). It would be interesting to analyze whether this mutant and others affected in vacuole biogenesis (Cui et al. 2018) exhibit evident alterations in the migration of the AT nuclei and cortex in their roots.

## Conclusions

A comprehensive investigation has been conducted into the distribution of euchromatin and constitutive heterochromatin in the roots of *A. thaliana*, in conjunction with the analysis of DNA replication. Our findings illustrate that chromatin reorganization is a highly dynamic process occurring during the distinct phases of mitosis and immediately following cytokinesis. Furthermore, we observed an intriguing phenomenon of nuclear migration in certain cells at the transition domain of the RAM region and towards the elongation zone of the root. This phenomenon is associated with vacuole biogenesis and appears to be coordinated between the epidermis and the cells of the underlying cortex. The results demonstrate the structural dissimilarities of the nucleus and its internal organization between disparate cell types (T and AT), and illustrate a correlation between these structural dissimilarities and the processes of cell division and cell differentiation that occur in the RAM region of *A. thaliana* roots. This constitutes an invaluable tool for the study of mutants affected in the coordination of growth and the investigation of the impact of stress factors on chromatin organization, which will be the focus of future studies.

## Supplementary Information

Below is the link to the electronic supplementary material.

Supplementary file1 (PDF 3701 KB)

Supplementary file2 (PPTX 47723 KB)

## Data Availability

All data generated or analyzed during this study are provided in this published article and its supplementary data files or it will be provided upon a reasonable request.
