# Supplementary material for: Connecting high-resolution 3D chromatin maps with cell division and cell differentiation at the root apical meristem
Source: Plant Cell Rep. 2024 Sep 16;43(10):232. doi: 10.1007/s00299-024-03322-8 (PMC11405483; doi:10.1007/s00299-024-03322-8)
Supplement: Supplementary file 1 — Supplementary file1 (PDF 3701 KB) [file 299_2024_3322_MOESM1_ESM.pdf]

Suppl. Figure S1

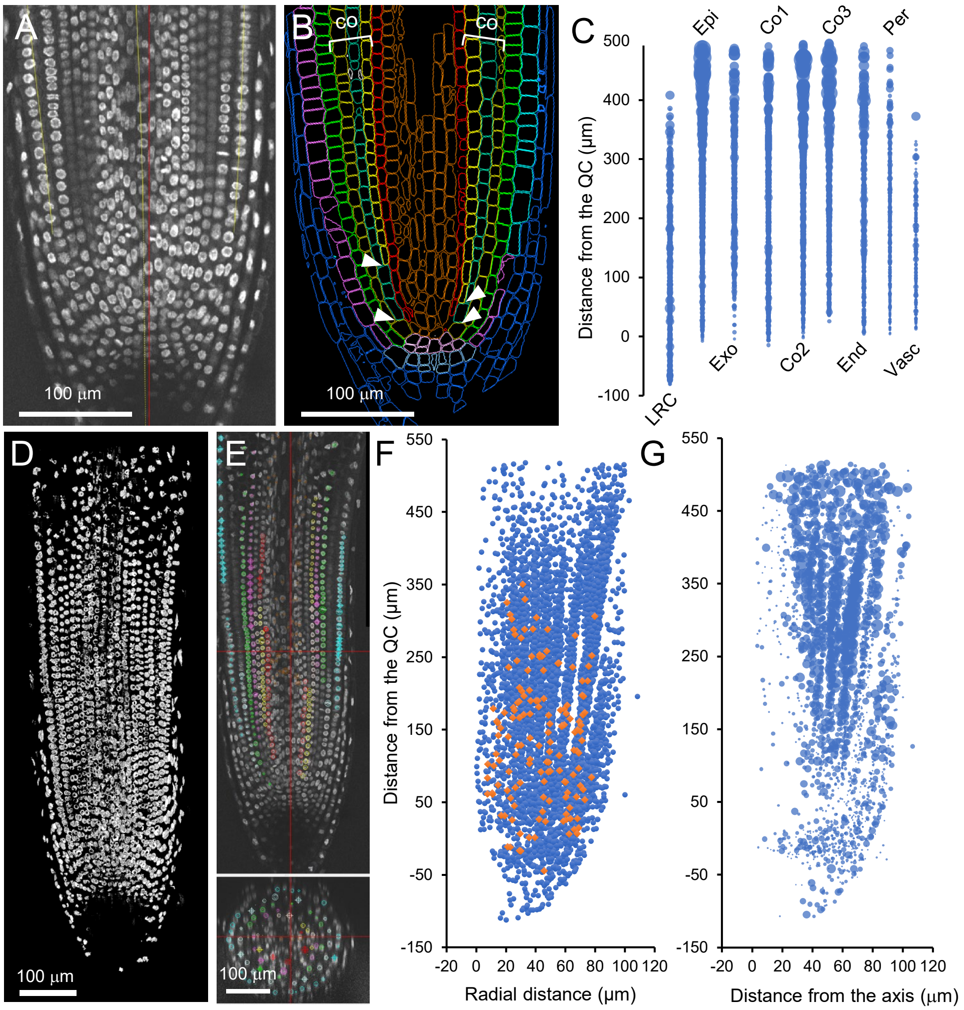

Figure S1. Cellular structure and nuclear heterogeneity in the RAM of *S. lycopersicum* var. M82. (A) DAPI labeling of a representative tomato root tip. (B) Cellular structure of the tomato root tip shown in A. Each color represents different tissue types (light blue, AT cells; magenta: T cells; light green, exodermis; dark yellow, blue and light yellow, C1, C2, and C3 cortex layers, respectively; red, endodermis; orange, inner pericycle and vascular layers). White arrows indicate asymmetric formative divisions in the cortex layer. (C) Bubble size indicated cell volume in the different tissue layers as regards their distance from the QC. LRC: lateral root cap, Epi: epidermis, Exo: exodermis, Co: cortex, End: endodermis, Per: pericycle, Vasc: vasculature. (D) Movie of whole root nuclei segmentation. (E) Nuclei have been detected and classified according to tissue type and fate as indicated in the main text; each color represents nuclei from same layer. Upper panel: longitudinal section, lower panel: cross-section. (F) Cell division map of the RAM along the longitudinal and radial axes of different layers (nuclei: blue; mitosis: orange). (G) Nuclei fluorescence plot as a function of distance from QC and radial root axis. Bubble size indicates intensity of DAPI fluorescence per nuclei.

Suppl. Figure S2

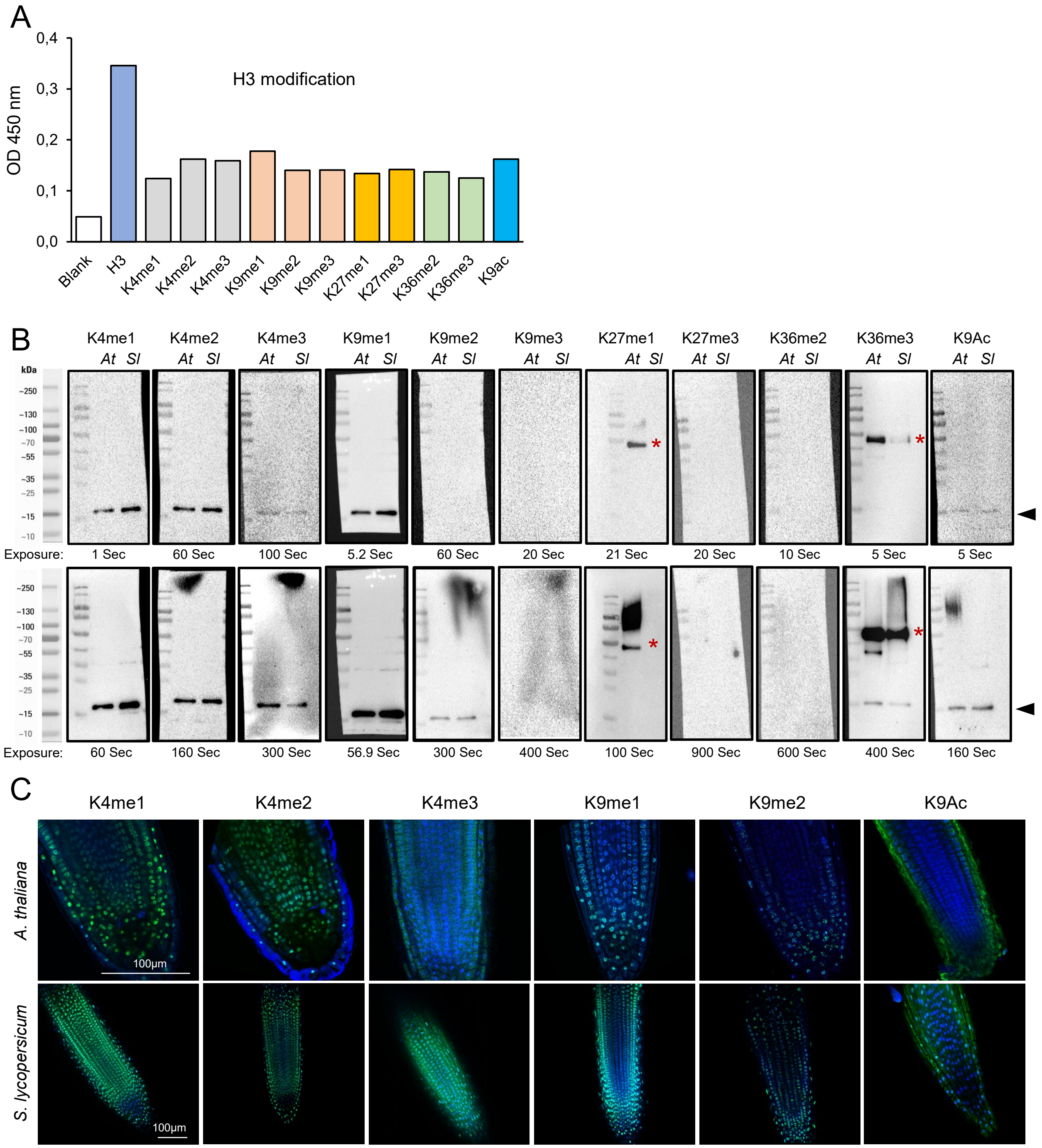

Figure S2. Evaluation of antibodies used for 3D chromatin analysis. (A) Quantification of some H3 marks using the EpiQuik Histone H3 Modification Multiplex Assay Kit (EpiGeneTek) from an extract of nuclei from 2-week-old *A. thaliana* Col-0 seedlings. (B) Western blotting with the selected antibodies in nuclear extracts from *A. thaliana* (*At*) and *S. lycopersicum* (*Sl*). Black arrowheads indicated the position of the expected protein bands in the gel. Non-specific protein bands are indicated with asterisks in red. (C) Antibodies against selected H3 tags were immunolocalized in the roots of *A. thaliana* and *S. lycopersicum*. The nuclei were counterstained with DAPI (blue).

Suppl. Figure S3

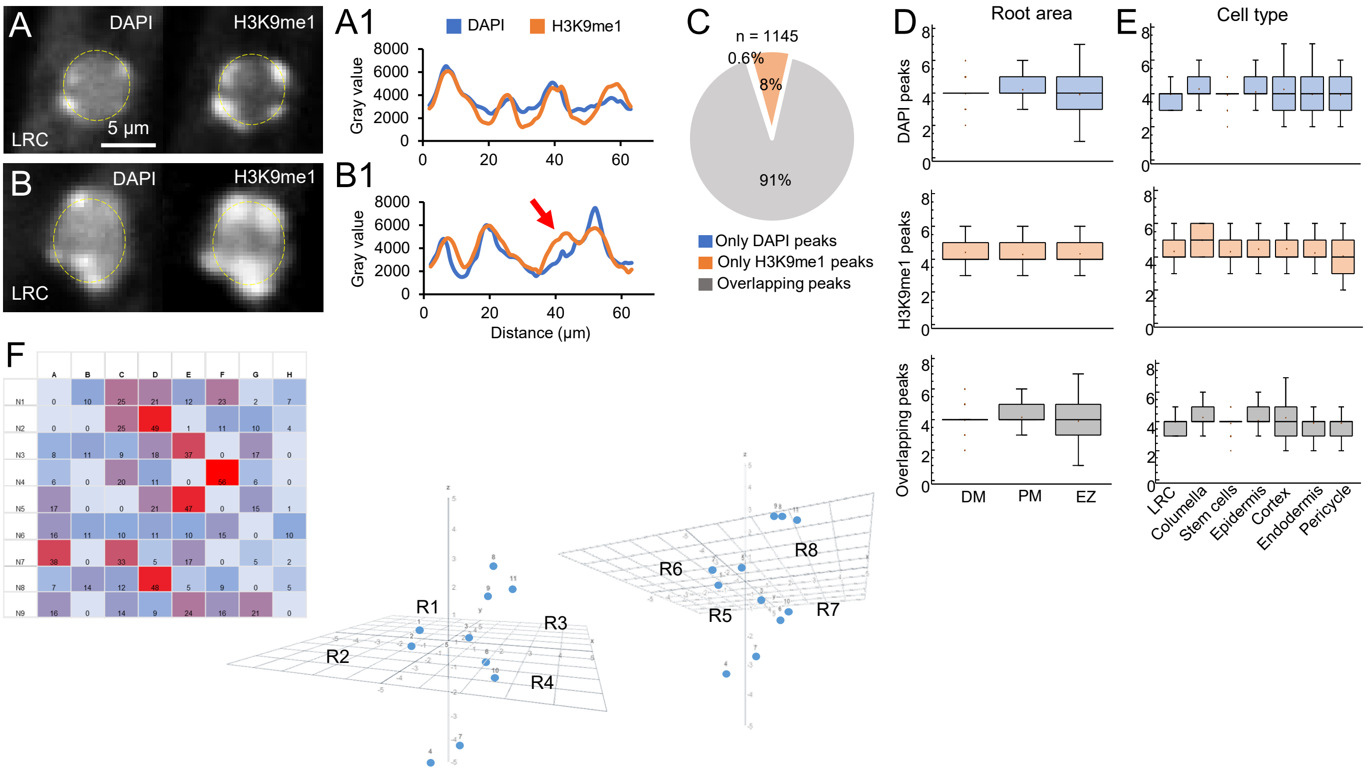

Figure S3. Analysis of the overlap between heterochromatin and DAPI chromocenters. (A-B) Intensity profiles of DAPI and H3K9me1 signals for representative nucleus of the LRC cells. Intensity profiles of DAPI and H3K9me1 signals for representative nucleus showing overlapping peaks between both signals (A, A1) and exclusive H3K9me1 peaks (B, B1). The nucleus periphery was manually defined for colocalization studies. (C) Proportion of overlapping peaks and exclusive peaks of DAPI or H3K9me1 in the analyzed nuclei (n = 1145). (D-E) The total number of peaks of DAPI, H3K9me1, or overlapping has been measured for different cell types (D) in different areas of the root (E). There are no significant differences in any of the cases. (F) 3D methodology used to study the spatial distribution of heterochromatin. The 3D object counter plugin in Fiji was used to obtain the coordinates (x, y, z) for each heterochromatin particle. These coordinates can be used to represent the particles in 3D space using tools such as MATH3D. The coordinate axis has been divided into 8 sectors (R1 to R8) to quantify the number of particles or fluorescence intensity in each region. The table displays data for 10 nuclei, indicating an evident asymmetry in the distribution of heterochromatin.

Suppl. Figure S4

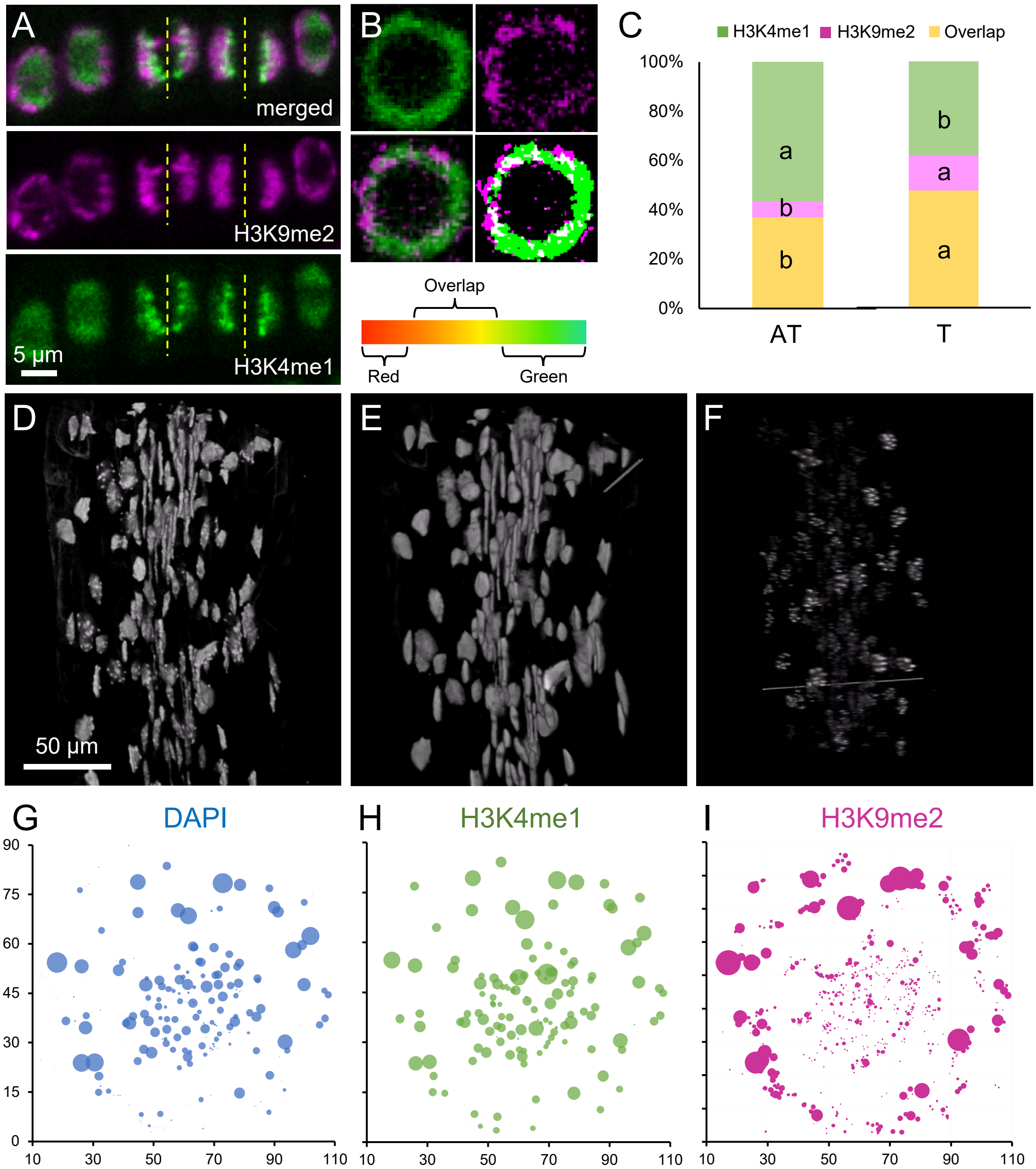

Figure S4. Colocalization studies of H3K4me1 and H3K9me2 in *A. thaliana* and tomato roots. (A) H3K9me2 (magenta) and H3K4me1 (green) localization in MZ of AT cells in tomato epidermis. Dashed yellow lines shows the equatorial plane of the cell division. (B) Approach used to study colocalization in single nuclei. Red and green channels were combined, and volume of the signal was assigned to different color ranges, as indicated. (C) Co-localization of H3K4me1 and H3K9me2 in AT and T cells of the MZ of the RAM in *A. thaliana*. Letters indicate statistically significant differences between cell types (*P*-value <0.01; LSD). (D-F) Triple staining with DAPI (D), H3K4me1 (E), and H3K9me2 (F) in the mature zone of a representative root. G, H, and I respectively show the vertical projection of individual nuclei from the DAPI, H3K4me1, and H3K9me2 immunolocalization shown above. Bubble size indicates fluorescence intensity of the indicated histone mark.

Suppl. Figure S5

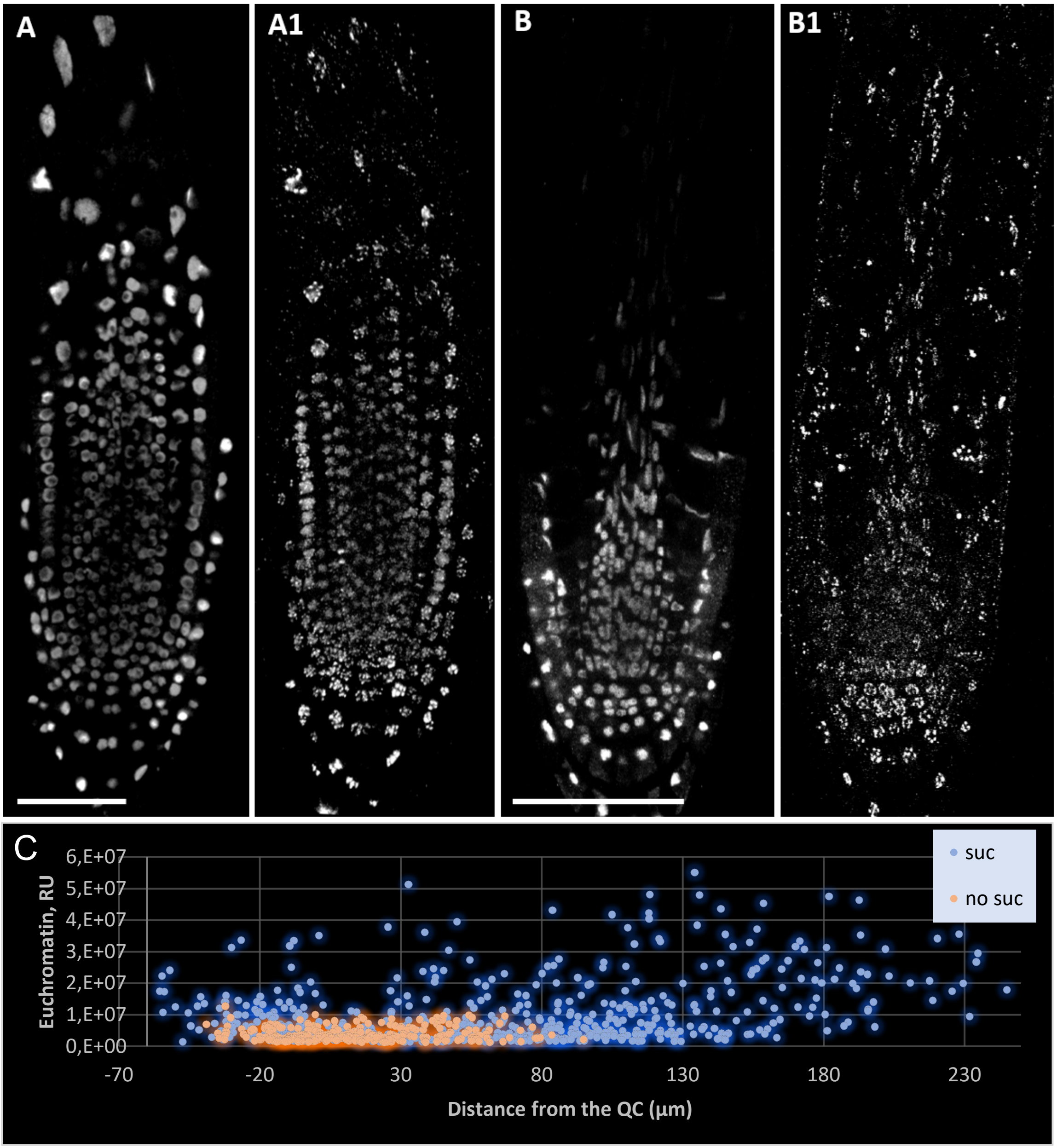

Figure S5. Chromatin features in response to sugar in *A. thaliana* roots. Seedlings were cultured in the dark for five days in the presence of 1% sucrose (A) or without sucrose (B). H3K4me1 (A, B); H3k9me2 (A1, B1). (C) Euchromatin fluorescence (H3K4me1 signal in relative units, RU) along the longitudinal root axis. Scale bars: 100 µm.

Suppl. Figure S6

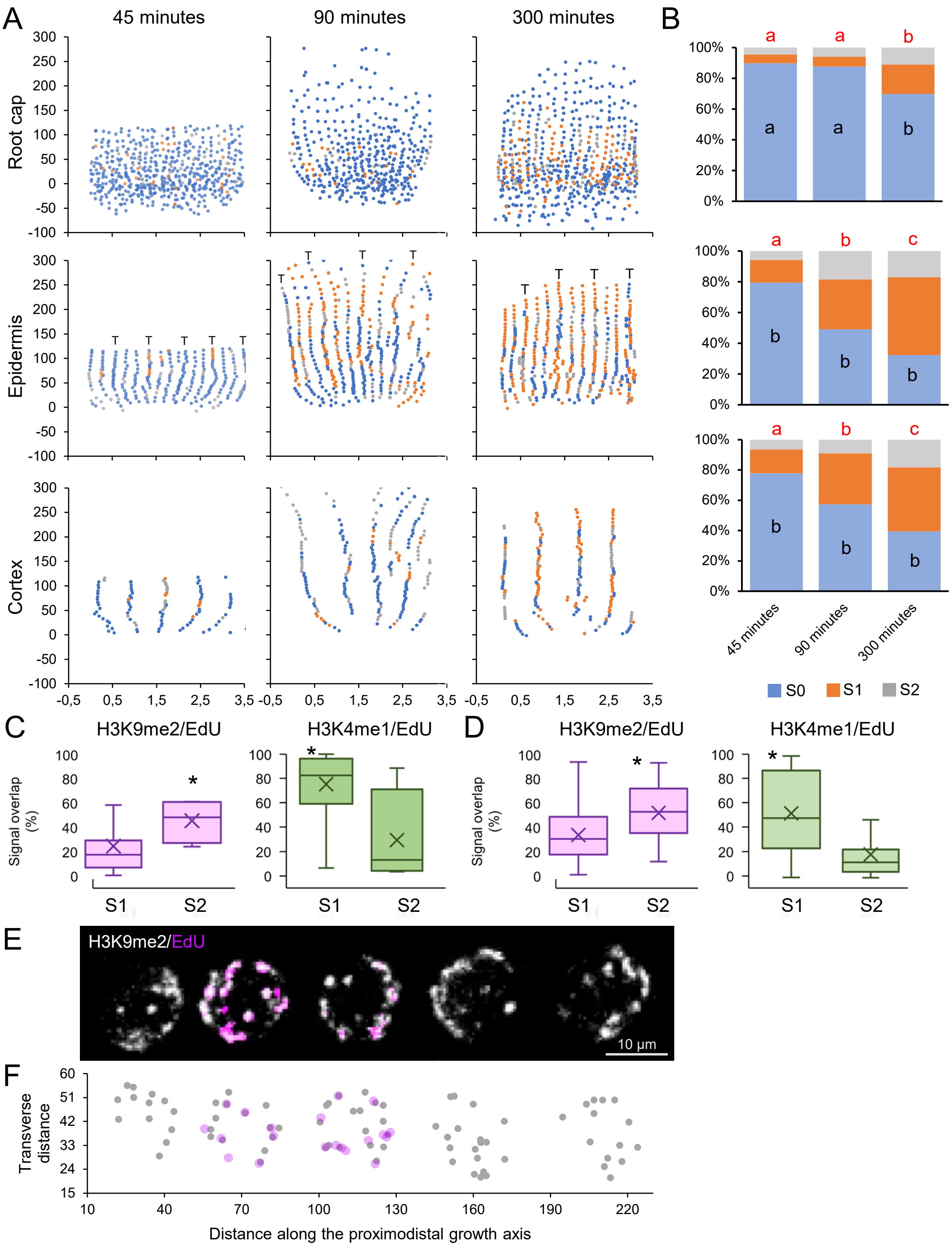

Figure S6. Analysis of replication states in whole root tissues during different time conditions. (A) Graphical representation of nuclei from *A. thaliana* root cap, epidermis (T indicated, AT the remaining cells), and cortex tissues were obtained using the iRocS program and represented on the X (distance from the QC, µm) and Y (radial distance, radians) coordinate axes, and assigned to different groups (S0, S1, or S2) based on their replication state after staining with EdU (45, 90, and 300 minutes). (B) Dynamics of EdU staining pattern at different time points depending on the cell type. The black letters indicate significant differences between different tissues for the same EdU incubation time. The red letters indicate statistical differences between different EdU incubation times within the same tissue. (P-value < 0.05; Chi-squared test). (C-D) Signal overlap between H3K9me2 or H3K4me1 with EdU have been measured in T (C) and AT (D) cells. Asterisks indicate significant differences between EdU patterns considered, S1 or S2. (E) Double staining and labelling with EdU (purple) and H3K9me2 (grey) in contiguous cortex nuclei in the MZ. Signal overlap is shown in white. (F) These nuclei were analyzed with the 3D object counter plugging and graphically represented using spatial coordinates. EdU spots are shown in purple, and H3K9me2 spots are depicted in grey.
