# Supplementary material for: Connecting high-resolution 3D chromatin maps with cell division and cell differentiation at the root apical meristem
Source: Plant Cell Rep. 2024 Sep 16;43(10):232. doi: 10.1007/s00299-024-03322-8 (PMC11405483; doi:10.1007/s00299-024-03322-8)
Supplement: Supplementary file 2 — Supplementary file2 (PPTX 47723 KB) [file 299_2024_3322_MOESM2_ESM.pptx]

## Slide 1
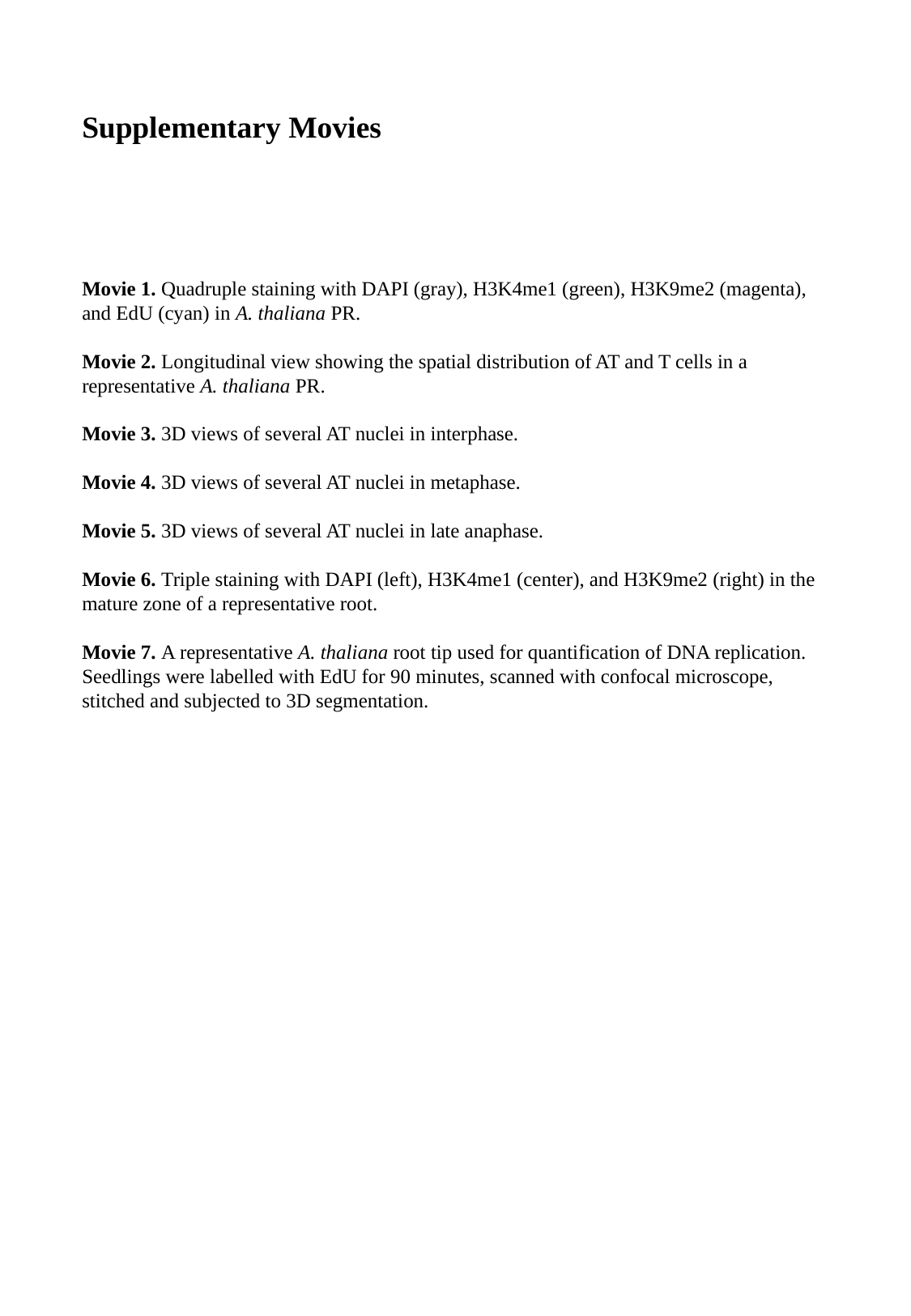

Supplementary Movies
Movie 1. Quadruple staining with DAPI (gray), H3K4me1 (green), H3K9me2 (magenta), and EdU (cyan) in A. thaliana PR.
Movie 2. Longitudinal view showing the spatial distribution of AT and T cells in a representative A. thaliana PR.
Movie 3. 3D views of several AT nuclei in interphase.
Movie 4. 3D views of several AT nuclei in metaphase.
Movie 5. 3D views of several AT nuclei in late anaphase.
Movie 6. Triple staining with DAPI (left), H3K4me1 (center), and H3K9me2 (right) in the mature zone of a representative root.
Movie 7. A representative A. thaliana root tip used for quantification of DNA replication. Seedlings were labelled with EdU for 90 minutes, scanned with confocal microscope, stitched and subjected to 3D segmentation.

## Slide 2
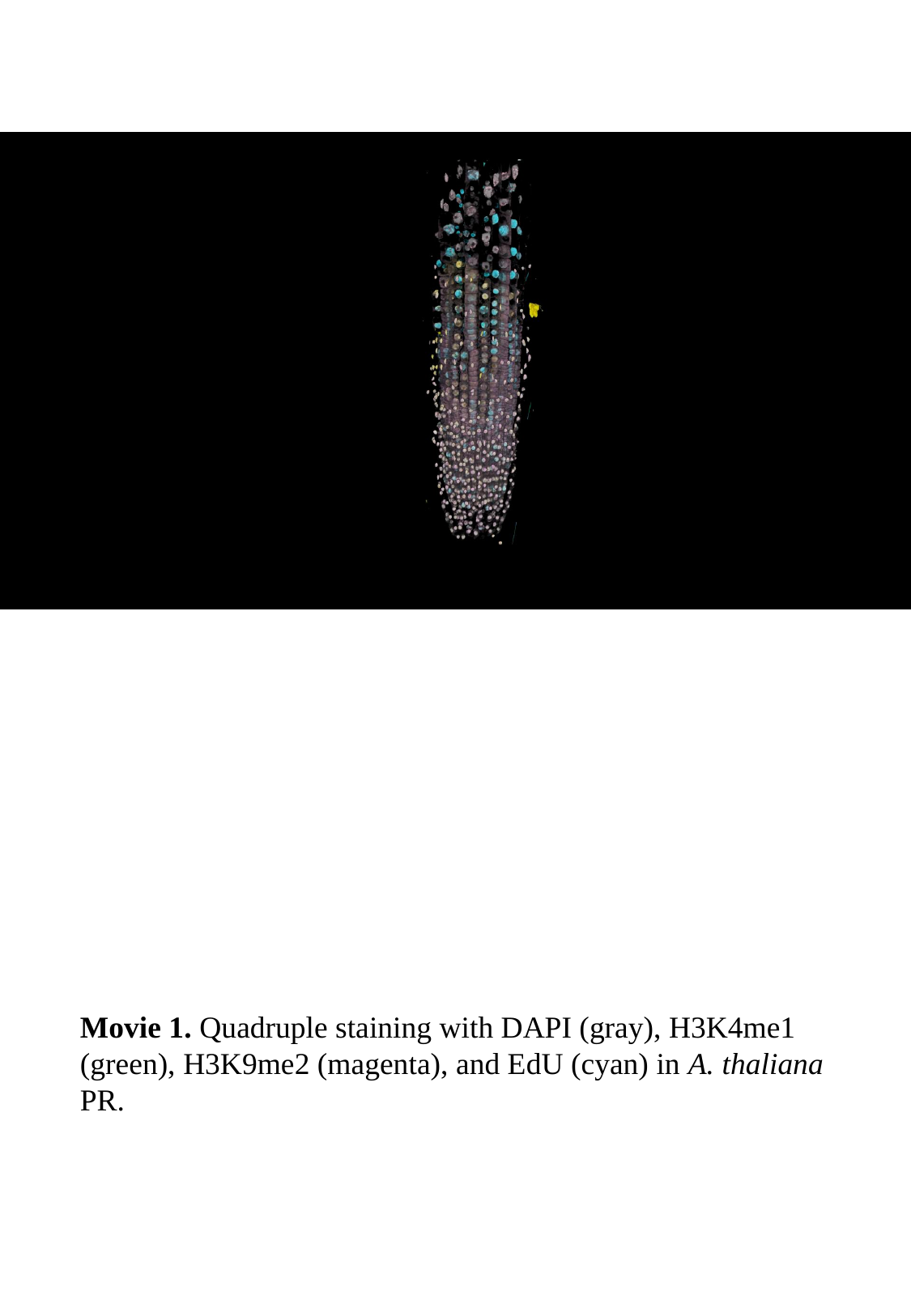

Movie 1. Quadruple staining with DAPI (gray), H3K4me1 (green), H3K9me2 (magenta), and EdU (cyan) in A. thaliana PR.

## Slide 3
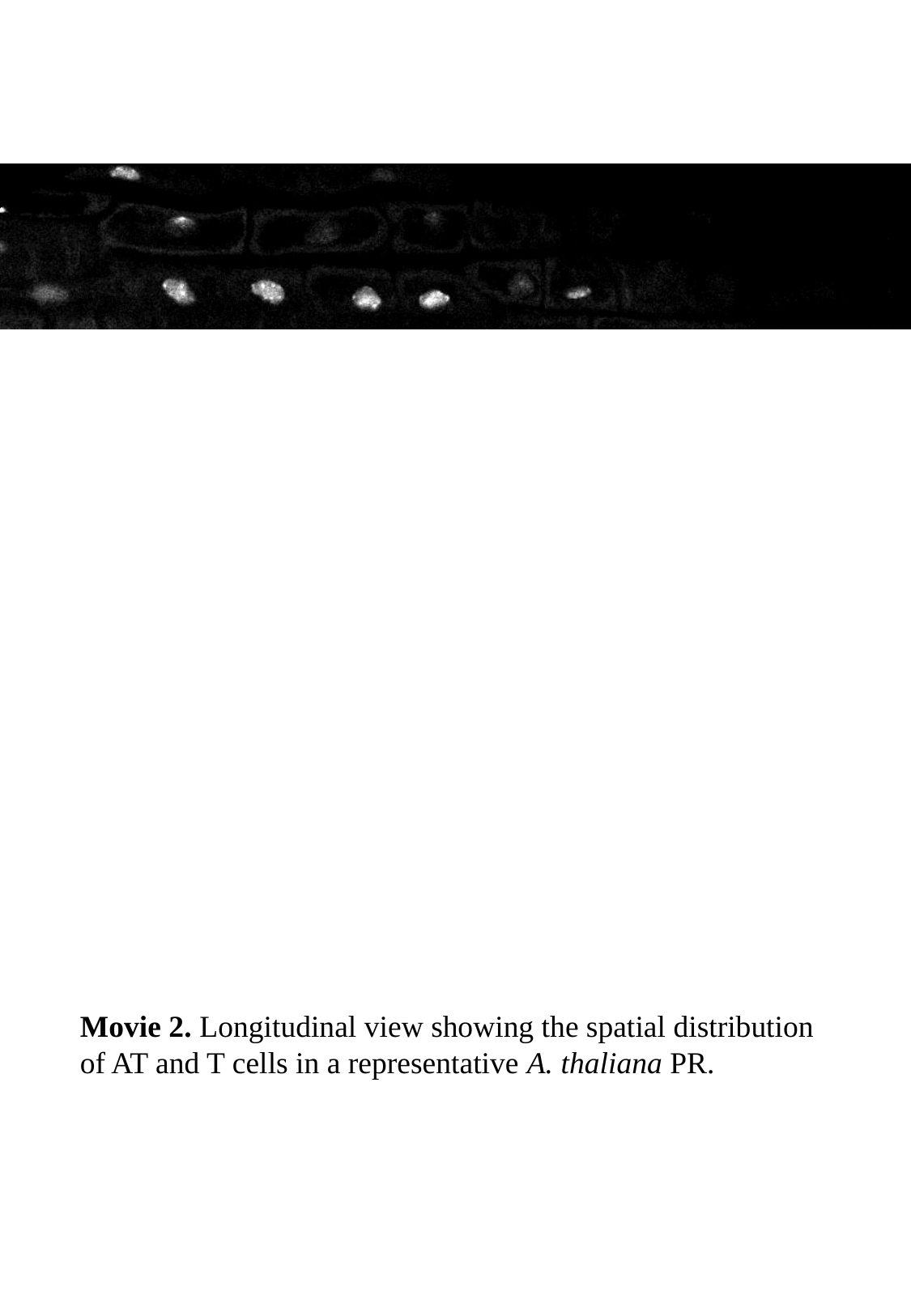

Movie 2. Longitudinal view showing the spatial distribution of AT and T cells in a representative A. thaliana PR.

## Slide 4
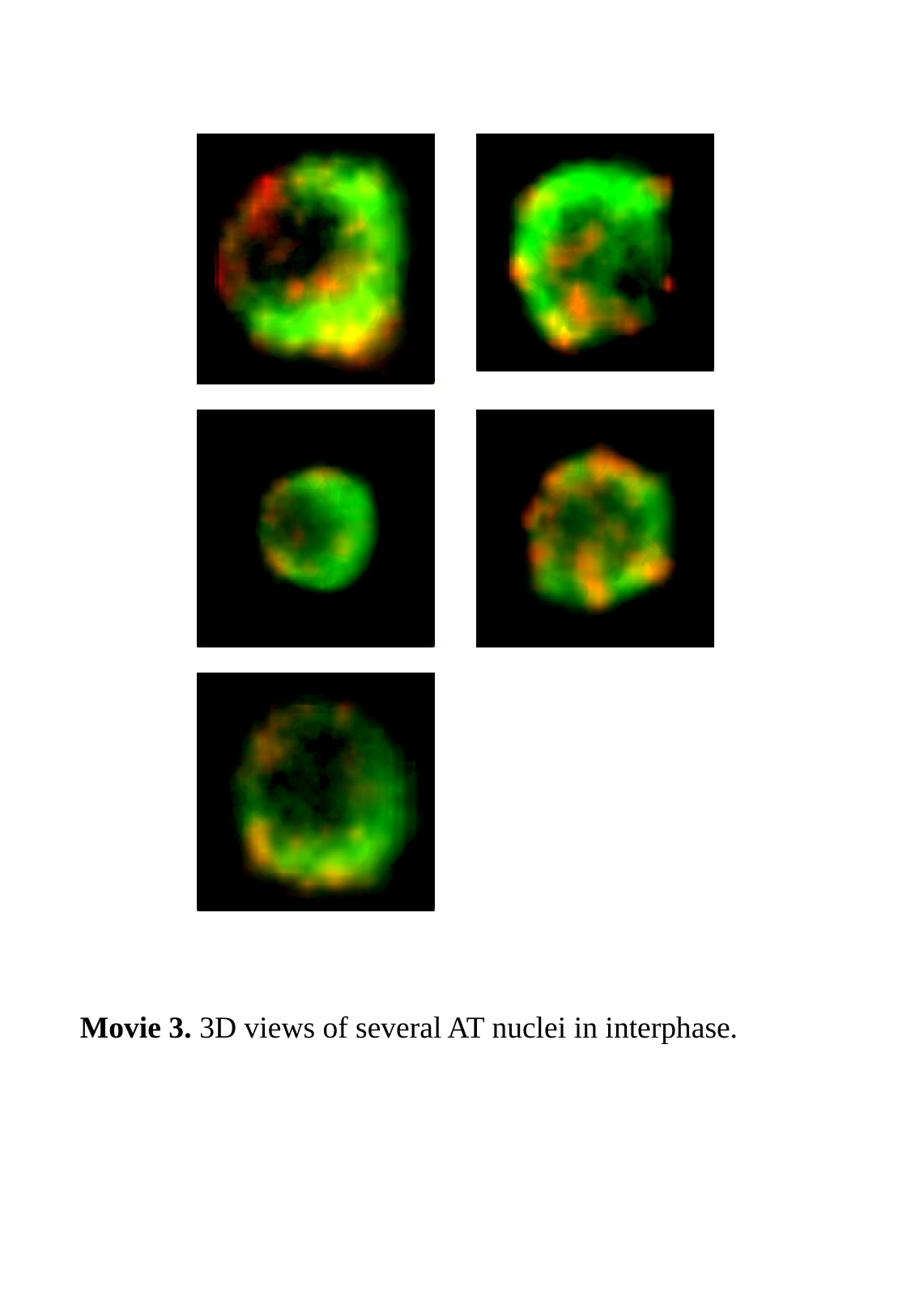

Movie 3. 3D views of several AT nuclei in interphase.

## Slide 5
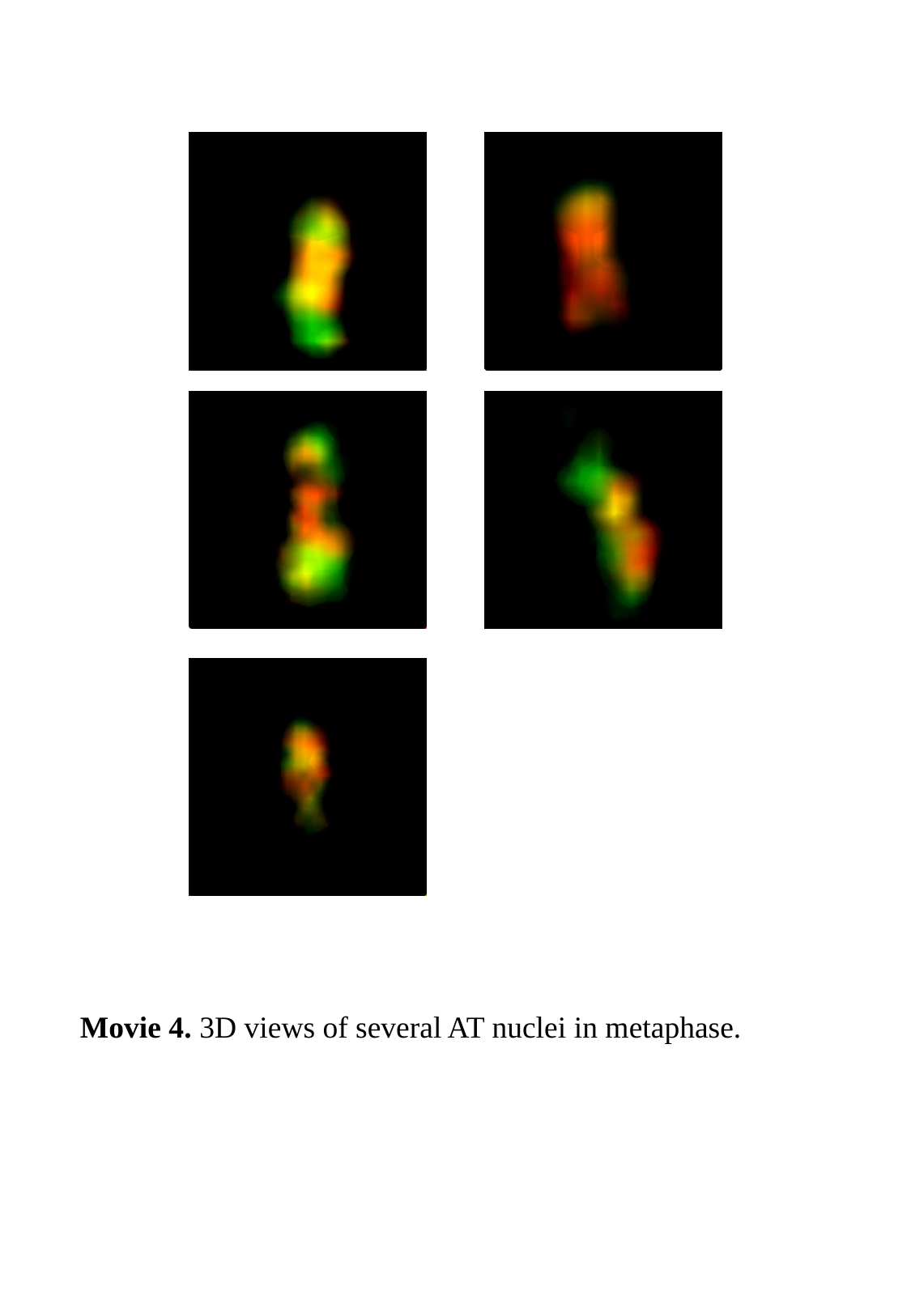

Movie 4. 3D views of several AT nuclei in metaphase.

## Slide 6
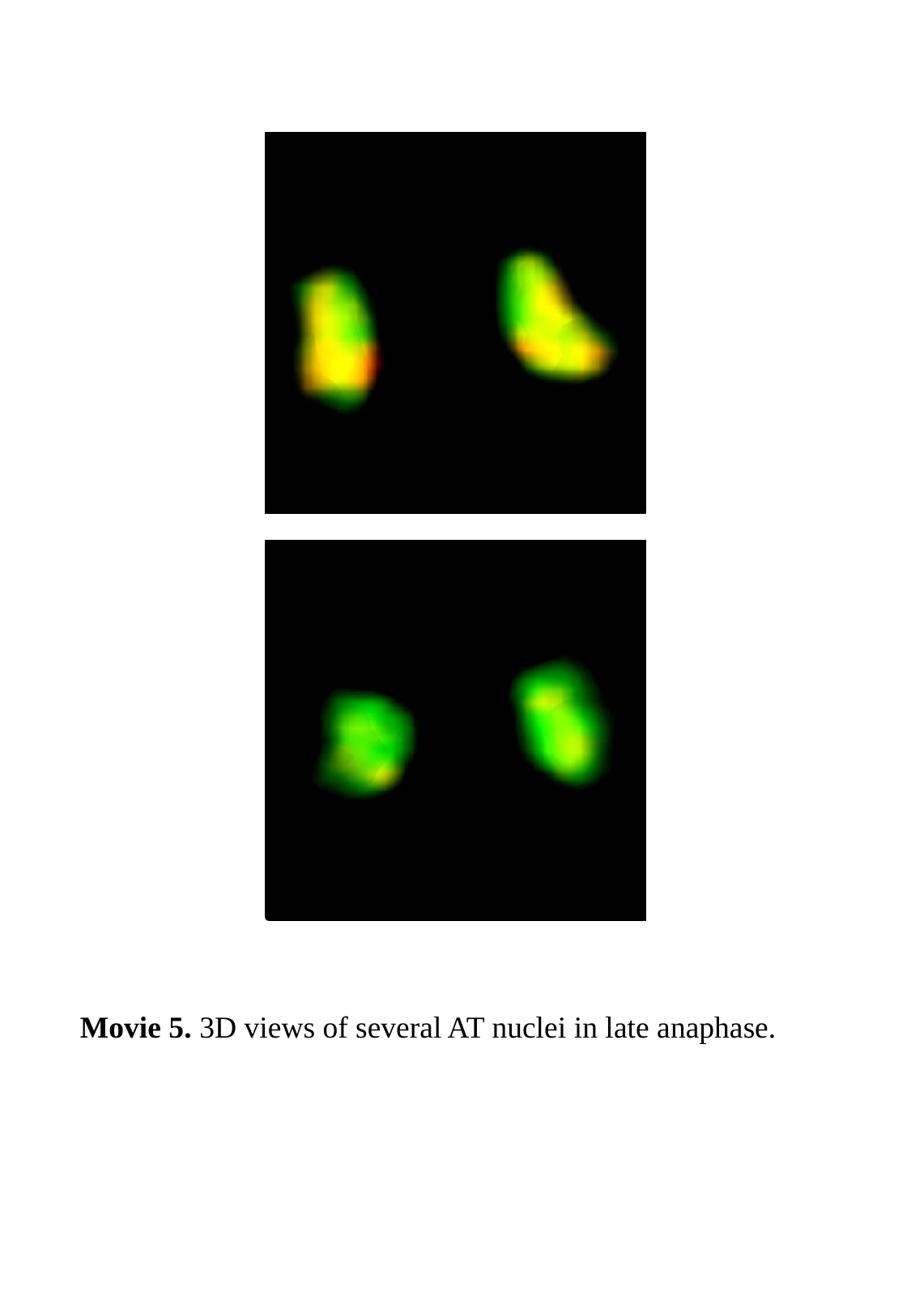

Movie 5. 3D views of several AT nuclei in late anaphase.

## Slide 7
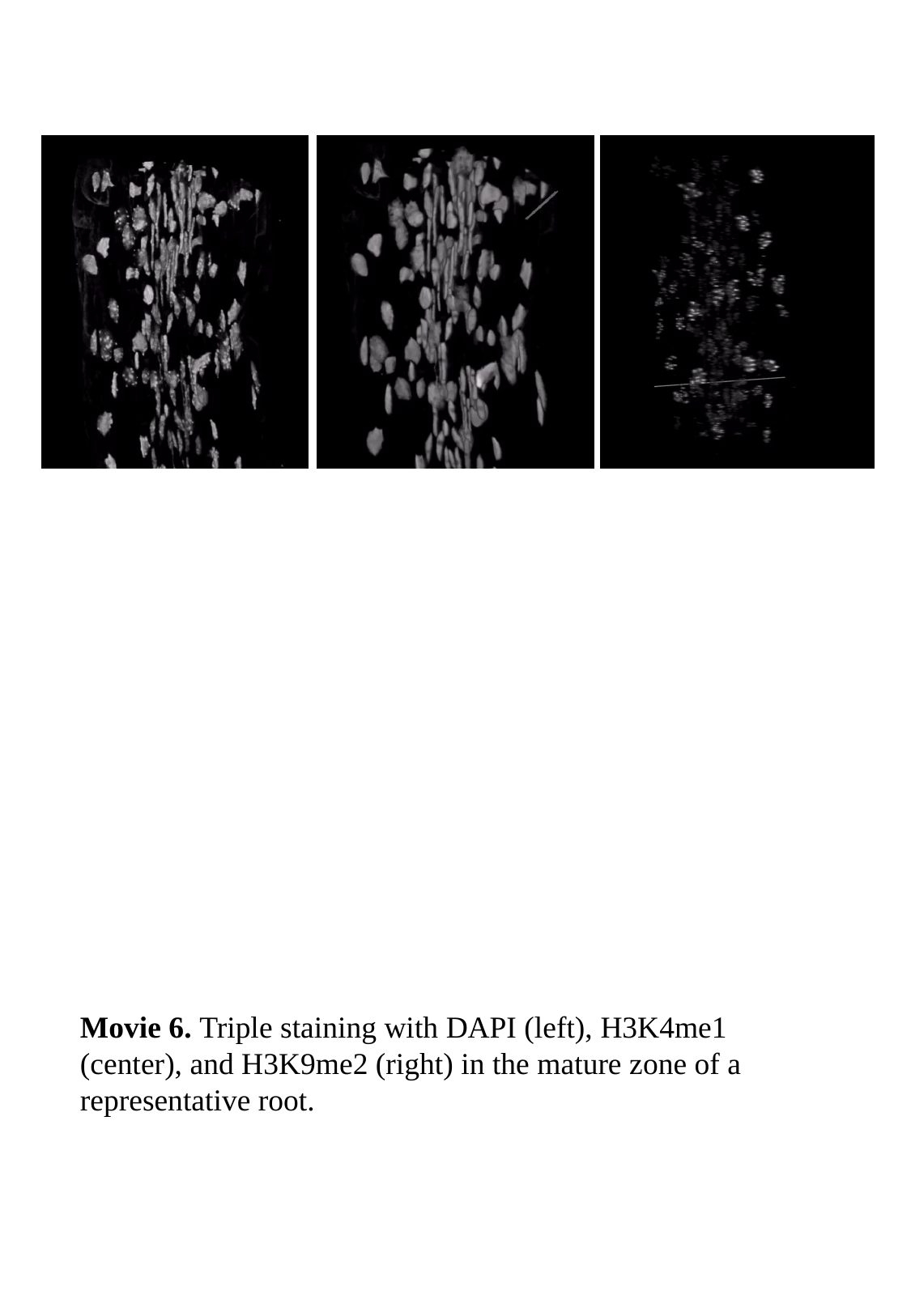

Movie 6. Triple staining with DAPI (left), H3K4me1 (center), and H3K9me2 (right) in the mature zone of a representative root.

## Slide 8
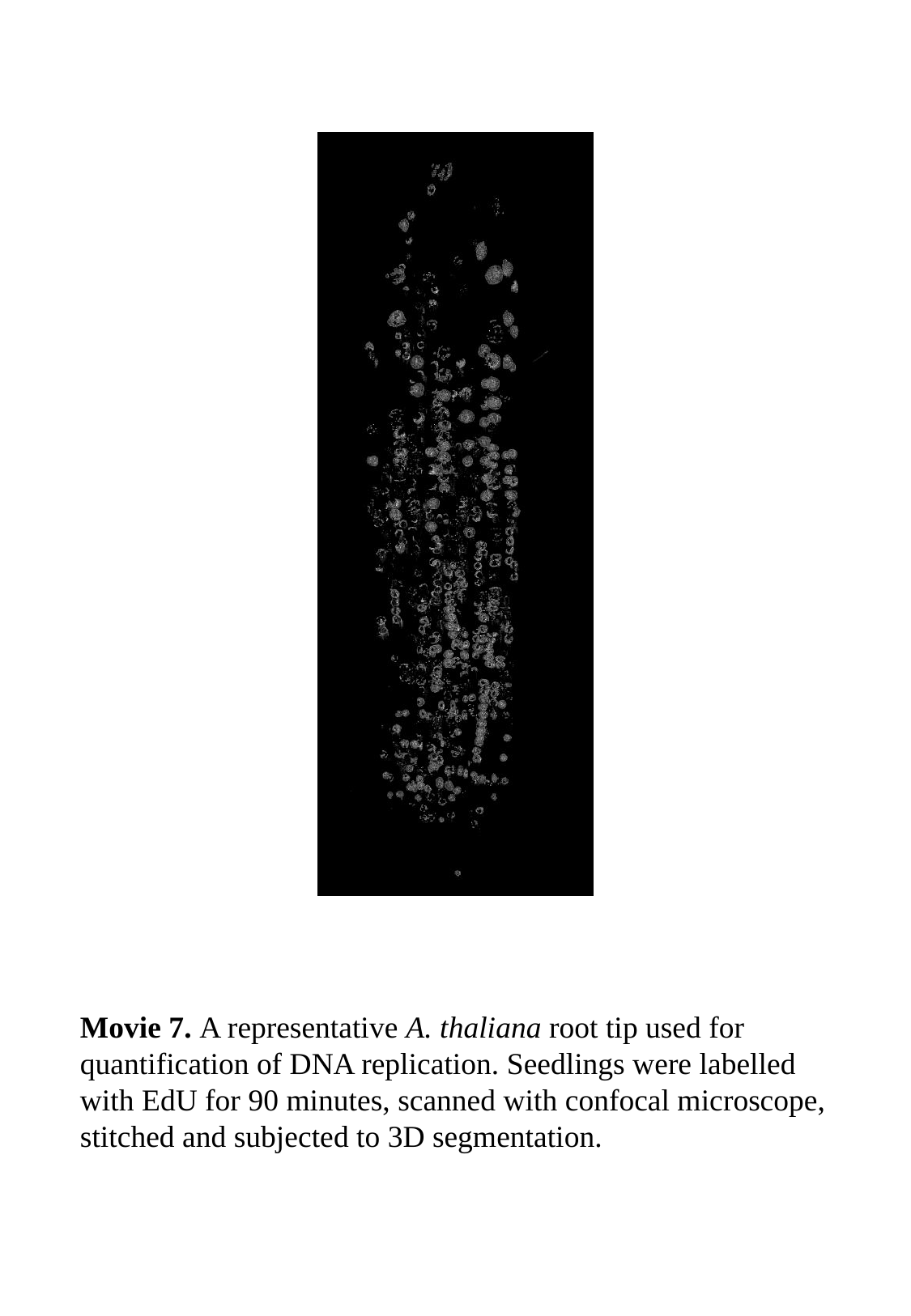

Movie 7. A representative A. thaliana root tip used for quantification of DNA replication. Seedlings were labelled with EdU for 90 minutes, scanned with confocal microscope, stitched and subjected to 3D segmentation.
